# Scoping review to assess the reach, effectiveness, and impact of government-funded, population-based physical activity initiatives in Australian adults

**DOI:** 10.3389/fspor.2025.1633086

**Published:** 2025-10-10

**Authors:** C. H. B. Dissanayaka Mudiyanselage, Stephanie E. Chappel, Sidney Irwin, Gabrielle Fisher, Alyson J. Crozier, Corneel Vandelanotte

**Affiliations:** ^1^ School of Health, Medical and Applied Sciences, Appleton Institute, Central Queensland University, Adelaide, SA, Australia; ^2^ School of Nursing and Midwifery, Deakin University, Burwood, VIC, Australia; ^3^ Centre for Quality and Patient Safety Research – Alfred Health Partnership, Institute for Health Transformation, Deakin University, Burwood, VIC, Australia; ^4^ Population Health Division, Preventive Health SA, Adelaide, SA, Australia; ^5^ Alliance for Research in Exercise, Nutrition and Physical Activity, Allied Health and Human Performance, University of South Australia, Adelaide, SA, Australia

**Keywords:** physical activity, government-funded, population-based, initiatives, effectiveness, reach, impact, Australia

## Abstract

**Introduction:**

In Australia, physical activity initiatives are often implemented by state and federal governments to enhance population-wide physical activity levels. Given the complexity and variability of government-funded physical activity programs, a scoping review is needed to synthesise the existing evidence and identify gaps in current initiatives. The aim of this review is to explore the reach, effectiveness, and impact of government-funded, population-based physical activity initiatives targeting Australian adults.

**Methods:**

This scoping review was conducted in accordance with the PRISMA extension for Scoping Reviews (PRISMA-ScR). PubMed, Scopus, Web of Science, MEDLINE, and ProQuest Public Health were searched for articles published between January 2000 and April 2024. Search terms included relevant terms surrounding the main topics of “physical activity,” “intervention,” “population-based,” “government-funded,” and “Australia.” Grey literature was collected from the websites of relevant organisations, health agencies of Australian states and territories, and other government departments. In addition, a manual search of references listed in primary sources was conducted to find journal articles missed during the database search. A narrative synthesis of included studies was conducted.

**Results:**

In total, 6,127 sources were identified, of which 71 were included in the final review. The peer-reviewed studies and grey literature evaluation reports identified physical activity initiatives across all Australian states and at the national level. Queensland and Victoria reported a greater number of physical activity interventions, strategies, and action plans compared to other states. The most common intervention strategies involved the use of digital platforms and a combination of multiple strategies.

**Discussion:**

While most of these initiatives increased physical activity, their overall reach to the broader Australian population was limited. The initiatives positively impacted individuals’ health (e.g., weight, mitigating chronic diseases) and well-being (e.g., developing social connections). Although these initiatives have demonstrated improvements in physical activity and community health and well-being, they have only reached a small fraction of the Australian population. This review highlights the need for a National Physical Activity Plan. While many states have published high-quality strategies and action plans, there is a pressing need for their actual implementation to assess effectiveness. Future research should focus on standardising evaluation frameworks and exploring strategies to enhance the sustainability and effectiveness of initiatives, particularly in diverse populations.

**Systematic Review Registration:**

https://osf.io/6aev4/registrations, identifier 6aev4.

## 1 Introduction

The health benefits of physical activity play a vital role in public health by preventing and managing chronic diseases and other health conditions (1, 2). Physical inactivity is associated with the development of many chronic diseases, such as cardiovascular diseases, type 2 diabetes, obesity, and several types of cancer globally (3). The World Health Organization (WHO) estimates that insufficient physical activity contributes to 3.2 million global deaths (4). Given the importance of physical activity, the WHO (5) developed the Global Action Plan on Physical Activity (GAPPA) to improve health and well-being, reduce healthcare costs, and support the economy. Using the GAPPA framework, governments have implemented and funded physical activity programs to increase community participation (6).

The Australian government has developed physical activity guidelines for different age groups to encourage regular physical activity and reduce sedentary behaviour (7, 8). While the national physical activity guidelines recommend a minimum of 150 minutes of moderate-intensity physical activity a week, recent estimates suggest that more than half of Australian adults (55%) do not meet these recommendations (9). In Australia, physical activity initiatives are implemented at both state and federal levels to enhance population-wide physical activity levels. Australian examples include the 10,000 steps program, which has a national reach despite receiving only state-based funding (10), as well as the *This Girl Can* campaign (11) and the *Get Active* program (12), both of which are state-level initiatives funded by the Victorian government. Although physical activity initiatives have been implemented in Australia for some time, the ways in which these programs are evaluated in terms of their reach, effectiveness, and impact are not always clear (13, 14).

Evaluating population-wide physical activity programs can be challenging due to the complexity of, and heterogeneity within, initiatives (15–18). For example, an initiative that targets school-aged children and aims to increase active play during recess differs vastly from one that targets older adults and promotes regular walking groups for cardiovascular health. These differences in target populations, goals, and methods require tailored evaluation approaches, making it difficult to compare or generalise outcomes. In Australia, evaluations of government-funded physical activity initiatives often face several gaps and challenges (19). One common issue is the use of a well-defined, comprehensive evaluation framework the outset of many initiatives (18). This can lead to inconsistent data collection methods and difficulties in comparing effectiveness across different programs (18). The omission of this key process also limits the ability to judge the value and translation of these initiatives, making it harder to determine their effectiveness and impact (15–18). In addition, most evaluations examine only short-term outcomes due to constraints in resources, time, and the complexity of tracking long-term behaviour change and health impacts (15, 20, 21). As such, establishing the long-term impact of government-delivered physical activity initiatives can be challenging.

Although population-based physical activity initiatives are implemented in Australia, limited knowledge exists regarding whether they are being evaluated, how evaluations are conducted, and what outcomes are achieved (22). As a result, we know little about the impact of these programs, which types are effective, and whether they provide good value for money. Therefore, a scoping review, which incorporates a comprehensive and systematic approach, can help to increase our understanding of the landscape of government-funded physical activity programs in Australia. A scoping review will serve to highlight areas that require attention, improvement, or further exploration (7), especially by considering not only peer-reviewed research articles but also grey literature such as evaluation reports and policy, strategy, and action documents produced by relevant government organisations (at the federal, state, and local council levels). Government policies, strategies, and action plans were included in the scoping review because they provide valuable insights into policy priorities, implementation frameworks, and strategic directions that shape the development and delivery of government-funded physical activity initiatives. While these documents do not provide details on reach, effectiveness, and impact, they can help distinguish between strategy and action intentions with the actual implementation and evaluation of physical activity initiatives. From herein, the term initiatives is used broadly throughout this review to refer to all potential funded physical activity programs, interventions, evaluations, policies, strategies, and action plans.

The aim of this scoping review is to explore the reach, effectiveness, and impact of government-funded, population-based physical activity initiatives targeting Australian adults. In particular, the research objectives are to (i) identify government-funded physical activity initiatives and their characteristics across Australia, and (ii) determine their reach, effectiveness, and impact on population-wide physical activity levels. This review has the potential to significantly impact public health and policy decisions by providing critical insights into government-funded, population-based physical activity initiatives and their impact on population physical activity levels and health.

## 2 Materials and methods

This scoping review was conducted according to the PRISMA extension for Scoping Reviews (PRISMA-ScR), which includes 20 essential items and two optional items (see Supplementary Table S1). The PRISMA flowchart was used to guide the study selection process (23). The protocol for this scoping review is registered on the Open Science Framework (https://doi.org/10.17605/OSF.IO/SHFJG) (24).

### 2.1 Search strategy and search terms

Electronic databases, including PubMed, Scopus, Web of Science, MEDLINE, and ProQuest Public Health, were searched on 2 April 2024. This scoping review utilised the PICOS framework (25), comprising population (i.e., Australian adults), intervention (i.e., government-funded physical activity initiatives or programs), comparison (not required due to the exploratory nature of scoping reviews), outcomes (i.e., reach, effectiveness, and impact), and study design (e.g., peer-reviewed studies and grey literature, including evaluations and reports) to create search terms. Five key search concepts were used: “physical activity,” “intervention,” “population-based,” “government-funded,” and “Australia.” The researchers collaborated with an experienced academic librarian at Central Queensland University, Australia, to develop comprehensive and relevant search terms. All search strategies are included in Supplementary Table S2. The search was restricted to publications from January 2000 to April 2024 to ensure that the review captured contemporary methods, trends, and initiatives relevant to current physical activity practices and policies. The search criteria were restricted to English-language, full-text, human studies involving adult populations (aged over 18), as well as journal articles, government reports, and official publications. A manual search of references listed in primary sources was conducted to find any relevant journal articles that may have been missed during the database search.

A grey literature search, using the same restrictions as the peer-reviewed literature search, was conducted by exploring websites of relevant organisations, such as preventive health agencies of Australian states and territories (e.g., Preventive Health SA, VicHealth, Queensland Health), health agencies of Australian states and territories (e.g., South Australian Department of Health and New South Wales Department of Health), other related state-level government departments (e.g., Department of Transport and Main Roads Queensland), and the Australian federal government, to find reports and policy documents related to physical activity initiatives. Key government organisations were also contacted to request access to internal reports on physical activity initiatives. In addition, a Google Advanced Search was performed using combinations of keywords such as “government-funded physical activity initiatives,” “physical activity strategy,” and “action plan,” alongside jurisdiction-specific terms (e.g., “South Australia,” “Australia,” and “state-level”).

### 2.2 Screening

After collecting relevant peer-reviewed articles from electronic databases, all data were exported to EndNote, where all duplicates were manually deleted. The remaining peer-reviewed articles were then imported into Covidence (https://www.covidence.org), which automatically detected and removed duplicates. Collected grey literature documents were downloaded from the internet and saved in PDF format in a folder. A list of document titles and their available online links was compiled in an Excel spreadsheet, with duplicates removed manually. The same inclusion and exclusion criteria were applied for peer-reviewed and grey literature, as presented in Table 1.

**Table 1:** Inclusion and exclusion criteria.

Inclusion criteria	Exclusion criteria
Government-funded physical activity initiatives	Studies or reports of government-funded initiatives that were not conducted in Australia
Conducted at the local, state, or federal/national level in Australia	Non-government-funded physical activity initiatives
Adult population (aged over 18)	Aged under 18
Written in English	Non-English
Population-based initiatives with the potential to reach a large number of people	Initiatives that do not have the potential to reach large groups of people
Published between January 2000 and 2024	Published before 2000
	Initiatives that did not focus on physical activity
	Review articles

A single author (CD) conducted the database search for relevant studies and collected grey literature. All screening was performed in duplicate to ensure accuracy (26). Before the main screening, a pilot screening of titles, abstracts, and full text was conducted for 10% of the peer-reviewed articles and grey literature by four authors (CD, SI, CV, and SEC). Subsequently, two authors (CD and SI) independently screened all titles, abstracts, and full texts to determine eligibility for inclusion in the review using Covidence software. Any disagreements between the two main reviewers (CD and SI) were resolved through discussion with two other team members (CV and SEC).

### 2.3 Data extraction

Initially, a pilot data extraction (10% of the sample) was conducted by a single author (CD), with subsequent verification of data accuracy and completeness by three other authors (CV, SEC, and SI). Following this, the final data extraction was conducted independently by a single author (CD) using three distinct extraction forms: one for peer-reviewed literature, one for grey literature evaluation documents, and one for grey literature strategy and action documents. The following data were extracted: “Name of the physical activity implementation or initiative,” “Summary of the program,” “Jurisdiction,” “Start year,” “End year,” “Type of physical activity,” “Type of Initiative,” and “Document type.”

For the peer-reviewed and grey literature evaluation documents, additional data extracted included “Evaluation type,” “Reach,” “Effectiveness,” and “Impact.” While reach is defined as the number and proportion of individuals who are willing to participate in a given initiative (27), this research expanded the concept of reach to include additional metrics such as organisational engagement, frequency of website views and usage, and step logins. Effectiveness was defined as “the degree to which something is successful in producing a desired result” (28). Given the diversity of physical activity initiatives, this scoping review did not only focus on program effectiveness but instead reported effectiveness based on the information available in the included documents. As such, organisational-level engagement and the effectiveness of digital platforms in promoting changes in community physical activity behaviours were examined as critical measures of effectiveness in addition to changes in physical activity levels resulting from the initiatives. This study defined impact as “a positive or negative, direct or indirect, intended or unintended change produced by an intervention” (29). In assessing the impact of physical activity initiatives, factors beyond changes in physical activity levels were also considered, including weight management, prevention of chronic disease, awareness of the initiatives, social connection, and the promotion of healthy behaviours.

For peer-reviewed studies only, the following variables were extracted: “Aims and objectives,” “Population and sample size,” and “Study design.” For the grey literature only, the following variables were also extracted: “Priority areas,” “Principles,” “Actions/strategies for physical activity,” and “Methods of measurement or evaluation.”

### 2.4 Collating, summarising, and reporting results

The extracted data from the included documents were compiled into a summary table, and a descriptive summary of the demographics of the included documents was developed. The findings were narratively analysed and presented to address the purpose of this scoping review. This analysis included the characteristics, reach, effectiveness, impact, and the implementation of strategy and action related to physical activity initiatives. A quality assessment of the included documents was not conducted, as this is not commonly done for scoping reviews (30).

## 3 Results

**Figure 1:**
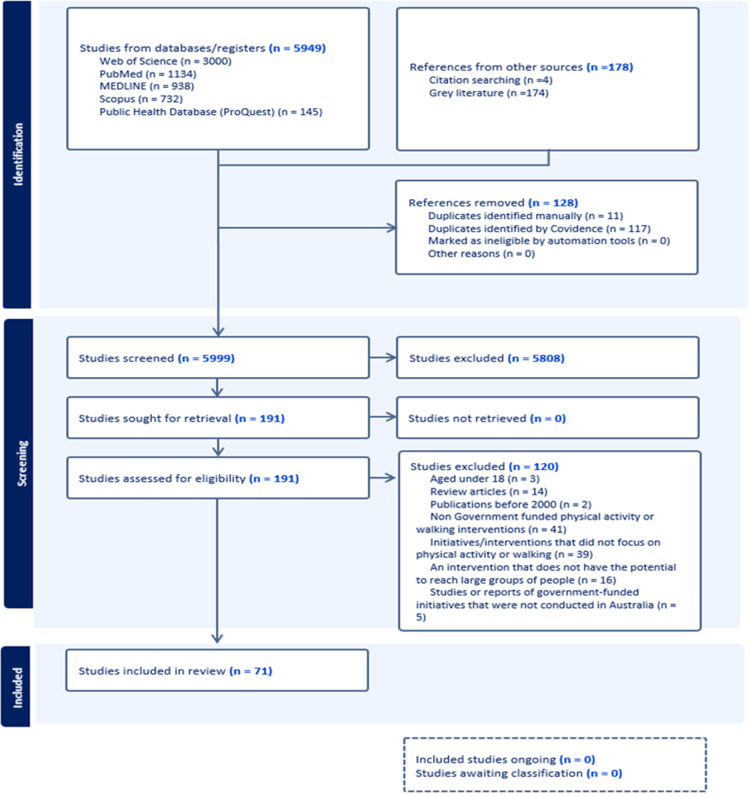
PRISMA diagram.

The initial database search for peer-reviewed sources retrieved 5,949 records. An additional 178 grey literature documents were identified, bringing the total number of documents to 6,127. After removing 128 duplicates, 5,999 records remained for screening, comprising 5,824 records from peer-reviewed sources and 174 from grey literature. Based on the inclusion and exclusion criteria, a full-text review was conducted on 191 records, including 138 peer-reviewed documents and 53 grey literature documents. Finally, 71 documents were included in the final review, consisting of 22 peer-reviewed documents and 49 grey literature documents (see Figure 1).

### 3.1 Characteristics of studies

An examination of peer-reviewed literature revealed 22 manuscripts that examined 11 government-funded, population-based physical activity initiatives implemented for adults in Australia, published between 2008 and 2023.

The grey literature findings were divided into two categories: evaluation reports and strategy and action documents. Twenty evaluation reports addressed 12 government-funded, population-based initiatives in Australia, with an additional 29 strategy and action documents identified. Table 2 presents the summarised characteristics of the included peer-reviewed studies, evaluation reports, and strategy and action documents.

**Table 2:** Summarised characteristics of included peer-reviewed studies, evaluation reports, and strategy and action documents.

Category	Specific characteristics	Count (%)		
		Peer-reviewed studies	Evaluation reports (grey literature)	Strategy and action documents (grey literature)
	Number of initiatives/strategies and action plans	11	12	23
	Number of records	22	20	29
Publication/initiative year	2000–2010	3 (14)	7 (35)	2 (7)
	2011–2020	13 (59)	10 (50)	16 (55)
	2021–2024	6 (27)	3 (15)	11 (38)
Jurisdiction	National	8 (36)	4 (20)	–
	New South Wales	5 (23)	2 (10)	4 (14)
	Victoria	3 (14)	5 (25)	7 (24)
	Tasmania	3 (14)	1 (5)	1 (3)
	Queensland	3 (14)	7 (35)	7 (24)
	Western Australia	–	–	3 (10)
	Northern Territory	–	–	1 (3)
	South Australia	–	1 (5)	6 (21)
Physical activity type	Walking and physical activity	19 (86)	13 (65)	18 (62)
	Cycling	1 (5)	3 (15)	7 (24)
	Transport related physical activity	2 (9)	–	–
	Walking and cycling	–	–	3 (10)
	Sports and walking	–	4 (20)	1 (3)
Intervention type	Website and mobile app initiatives	6 (27)	1 (5)	–
	Promotional programs and educational initiatives	6 (27)	–	1 (3)
	Social marketing and social media campaign	2 (9)	2 (10)	–
	Telephone coaching	2 (9)	–	–
	Grants and incentives to facilitate program initiatives	3 (14)	–	–
	Policies, strategies, and action plans	2 (9)	4 (20)	28 (97)
	Evaluation sessions	1 (5)	–	–
	Provide active space and networking	–	2 (10)	–
	Multiple initiatives	–	11 (55)	–
Initiative period	6 months or less than 6 months	10 (45)	–	–
	6 months to 1 year	1 (5)	–	–
	1–3 years	7 (32)	10 (50)	9 (31)
	Over 3 years	3 (14)	10 (50)	20 (69)
	Not applicable	1 (5)	–	–
Documentation type	Annual reports and reviews	–	–	6 (21)
	Evaluations	10 (45)	13 (65)	7 (24)
	Monitoring framework	–	–	1 (3)
	Other methods (data collection)	–	–	4 (14)
	Not specified	12 (55)	7 (35)	11 (38)

### 3.2 Peer-reviewed studies

#### 3.2.1 Characteristics

As presented in Table 2, among the 22 manuscripts analysed, eight (36%) were conducted at the national level (31–38), while five (23%) were specific to New South Wales (NSW). Three studies each were conducted in Victoria (14%), Tasmania (14%), and Queensland (14%), respectively. The majority of research (19 articles; 86%) concentrated on walking and general physical activity initiatives (31–35, 37–50). Three initiatives utilised website and mobile app approaches (31–34, 39, 41–43), while six initiatives employed promotional programs and educational approaches (36, 37, 40, 46, 48, 49). Many of the studies (45%) implemented their programs within 6 months or less (33, 38, 39, 42–44, 46, 48, 51, 52), while several extended over 1–3 years (32%) (34, 36, 40, 41, 44, 47). The included studies used different types of evaluations such as process, impact, and outcome evaluations (32, 39–41, 43, 47–49, 52), with 12 (55%) studies not specifying their evaluation methods (31, 33–38, 42, 44–46, 50, 52). The smallest sample size was a qualitative study including 35 participants (37), whereas the largest sample encompassed 425,000 participants (32). Additional information on the characteristics of peer-reviewed documents is provided in Supplementary Tables S3 and S4.

#### 3.2.2 Reach

Although all physical activity initiatives aimed to reach large groups of people, eight studies (36%) examined these initiatives with a small sample of participants prior to large-scale implementation, considering various demographics, intervention types, and study designs (34, 37, 44, 47, 48, 50–52). These studies included a minimum of 78 participants and a maximum of 371 participants per initiative. The remaining 13 studies (59%) implemented large-group physical activity initiatives, with participant numbers ranging from 1,695 to 425,000 (31–33, 35, 36, 38, 40–43, 45, 46, 49). Three of the 11 initiatives (i.e., the 10,000 steps program, Get Healthy at Work, and VicHealth MetroACTIVE demonstration grant program) achieved significant organisational-level physical activity engagement, reaching between 3 and 486 organisations (40, 44, 47).

#### 3.2.3 Effectiveness

Many of the included studies demonstrated significant improvements in physical activity levels among participants, with the proportion of participants meeting physical activity guidelines increasing by 11%–16% across studies. Across the included studies, the average weekly physical activity increased from 150 to 431.5 minutes, suggesting that the interventions were considerably effective. In some cases, participants more than doubled their baseline activity levels (31–33, 36–38, 40–43, 46, 48, 49, 52). However, 14% of the studies reported limited effectiveness (31, 35, 52), and another 14% found no significant effects (33, 35, 46). The limited effectiveness observed in some initiatives was evident during the COVID-19 pandemic, with average weekly steps reducing from 3% to 14% less steps taken (31, 52). In addition, certain personal factors were associated with a greater likelihood of continued engagement in web-based physical activity programs, including being an older adult, male, and a non-Australian participant (35).

#### 3.2.4 Impact

Government-funded physical activity initiatives demonstrated a measurable impact on the Australian community. Five studies (23%) indicated that these programs contributed to maintaining a healthy body weight, with participants experiencing an average weight reduction of 1.5–3 kg following the intervention period (39, 41, 45, 46, 48). In addition, two studies suggest that these initiatives indirectly helped mitigate chronic diseases (37, 45). Several initiatives showed notable improvements in healthy behaviours, such as healthy eating habits (*p* = 0.004) (40). Awareness of the initiatives improved moderately, ranging from 66% to 73% (32, 42). While one study (5%) also reported improvements in mental well-being (e.g., reducing depression and increasing happiness, calmness, and self-esteem) and social connections as a result of the initiative (49). Furthermore, some initiatives fostered greater community engagement and collaboration among local organisations, further strengthening their effectiveness (32, 42, 47, 48). See Supplementary Table S5 for more details on the reach, effectiveness, and impact of government-funded physical activity initiatives, as documented in peer-reviewed studies.

### 3.3 Grey literature

#### 3.3.1 Characteristics

The grey literature included two main types of documents: evaluation reports and strategy and action plans. Analysis of these sources identified that most evaluation reports (55%) and strategy and action documents (55%) related to physical activity initiatives were initiated between 2011 and 2020. Queensland was the most predominant source, contributing 35% of evaluation reports (53–59) and 24% of strategy and action documents (60–66). Victoria followed, contributing 25% of evaluation reports (67–71) and 24% of strategy and action documents (72–78). South Australia has emerged as a key player in physical activity initiatives, introducing five strategy and action plans since 2021 (79–84). Increasing walking and physical activity levels were the most commonly cited goals across all grey literature initiatives [65% of evaluation reports (53–56, 67, 71, 85–90) and 62% of strategy and action documents (65, 66, 72, 73, 77–80, 82–84, 91–97)]. Evaluation reports indicated that four physical activity initiatives employed a combination of multiple approaches, such as telephone coaching, mass media campaigns, training sessions, and awareness programs (54, 55, 68–71, 88–90, 98). Half of the evaluation documents indicated program initiatives lasting from 1 to 33 years (53, 56–59, 71, 86, 88–90), while an equal proportion reported initiatives lasting beyond 3 years (53, 54, 67–70, 85, 87, 98, 99). Furthermore, 69% of strategy and action documents revealed that the implementation period extended beyond 3 years, reflecting the long-term (10 years) establishment of many strategies (60, 61, 65, 72, 74–79, 83, 91–96, 100, 101). Supplementary Tables S6 and S8 provide further details of the characteristics of government-funded physical activity initiatives identified in the grey literature.

#### 3.3.2 Reach

Grey literature evaluation reports indicated that government-funded, population-based physical activity initiatives successfully engaged diverse demographic groups, including rural populations (69, 98), First Nations people (67), and disadvantaged communities (98). One initiative targeting First Nations populations reported that their total proportion of participation in the physical activity initiative had increased from 2% to 5% (67). Furthermore, over 340,000 women were described as being inspired to participate in physical activity in the community through the “This Girl Can Victoria” initiative (69, 70, 86). Two initiatives focused on participant engagement and completion rates, with program completion ranging from 135 to 9,051 individuals (68, 88, 89, 97). Four initiatives achieved broad population engagement, with participation ranging from 2,000 to 11,300 individuals during the implementation period (54, 55, 68, 71, 87, 89, 90). In addition, three initiatives actively promoted physical activity campaigns on social media, generating up to 2 million campaign views and between 5,866 and 75,000 website visits (67, 70, 86, 87). Moreover, the initiatives reached their target population through local organisations’ partnerships with councils (*n* = 10) (71, 86), stakeholder engagement (89), and implementation of strategy and action plans, such as constructing 91 km of bicycle riding infrastructure (56–59).

#### 3.3.3 Effectiveness

Findings from the evaluation reports indicated improvements in physical activity levels across most initiatives, with 34%–80% of participants meeting national physical activity recommendations at follow-up and reported weekly activity increasing by 223–306 minutes compared to baseline (54, 88, 89, 97). One initiative, however, showed a lower rate of improvement, with the percentage of walking trips increasing by only 1% (from 9% to 10.1%) and the number of trips under 1 km walks increasing slightly from 59% to 60% (40). Another initiative reported strong retention, with over 77% of participants remaining active after 6 months and 78% still participating after 3 years (54). Similarly, group walking programs demonstrated strong retention, with over 80% of participants remaining active after 6 months and more than 50% maintaining engagement after 3 years (54, 55). Interestingly, two initiatives reported a 5% increase in female participation in specific activities (e.g., bicycling) and a rise in physical activity participation among culturally diverse women, from 37% to 52% (54, 69, 70). Three initiatives enhanced participants’ confidence in engaging in physical activity, with 75% reporting sustained physical activity engagement (69, 85, 99). Finally, the Queensland cycling strategy showed that the proportion of Queenslanders who ride a bike at least once a year has remained unchanged in the 2 years following its launch, indicating slow progress in participant engagement in cycling activities post-implementation (57).

#### 3.3.4 Impact

The findings from the grey literature identified the multifaceted impacts of government-funded physical activity initiatives. Five initiatives reported improvements in general health outcomes, including reductions in Type 2 diabetes, as well as enhanced community engagement, social skills, and the creation of supportive networks that foster collaboration and shared goals (53, 54, 67, 86, 98). Two initiatives resulted in physical health benefits, including weight loss (ranging from 3% to 10% reduction in body weight) and reductions in waist circumference (averaging 3–5.5 cm) (67, 68, 88–90). Finally, two documents reported that physical activity initiatives were perceived to be cost-effective among dementia populations and a workplace settings (53, 85). Additional information on the reach, effectiveness, and impact of government-funded physical activity initiatives, as reported in grey literature, can be found in Supplementary Table S7.

#### 3.3.5 Strategy and action plan documents

Analysis of the strategy and action plan documents related to government-funded physical activity initiatives in Australia identified four key areas of implementation: (1) encouraging walking and physical activity to improve population health behaviours (61, 62, 66, 72, 77, 79–81, 92, 94, 95, 101); (2) building safe environments and developing infrastructure that support active lifestyles, such as pedestrian pathways, walkable places, and recreational facilities (60, 61, 66, 72, 76, 77, 80, 84, 91, 92, 94, 95); (3) prioritising strategic management, policy, advocacy, and planning (63–65, 73–76, 79, 80, 83, 91, 92, 95, 97, 100); and (4) enhancing accessibility and fostering a culture of physical activity within communities (92). Ten (34%) strategy and action documents highlighted certain priority areas, which included active transport, health and recreation, safety, networking of stakeholders and community, investments and infrastructure development, and marketing and economic development (60, 61, 63, 65, 72, 75, 76, 81, 93, 94, 100, 101). Most strategy and action documents upheld the principles of diversity, equity, accessibility, effectiveness, advocacy, and adaptability (66, 72–75, 77, 92, 97, 100). Further details on the strategies and actions of government-funded physical activity initiatives, as documented in grey literature, are provided in Supplementary Table S9.

## 4 Discussion

This scoping review explored the reach, effectiveness, and impact of government-funded, population-based physical activity initiatives in Australia. The findings identified that state and federal Australian government agencies have implemented a diverse range of initiatives aimed at increasing physical activity among adult populations across the nation. Notably, Queensland and Victoria contributed a greater number of initiatives/strategies than other Australian states. Moreover, many of these initiatives successfully engaged broad audiences, demonstrating their potential to reach diverse populations. The findings identified that positive effects on participants' physical activity levels occurred during and after implementation of these physical activity initiatives. In addition, peer-reviewed articles and grey literature documents indicated that government-funded physical activity initiatives had multifaceted impacts, including fostering participants' confidence, promoting healthy lifestyles, enhancing community engagement and social skills, improving mental health outcomes (e.g., reducing depression and increasing happiness, calmness, self-esteem), and encouraging healthier behaviours in workplace settings, ultimately leading to increased productivity and overall well-being.

This scoping review identified variability in the effectiveness of government-funded physical activity initiatives. Most physical activity initiatives showed positive effects on physical activity levels among participants during and after implementation. These findings align with other studies demonstrating effectiveness across different program characteristics, such as initiative methods, target populations, and health outcomes (21, 102–105). However, five initiatives with no obvious similarities reported minimal or no significant effectiveness in promoting physical activity engagement or meeting the physical activity guidelines. These limitations have been discussed in previous research and may be explained by socioeconomic barriers, limited accessibility, and cultural or social factors (106–108). This scoping review also highlights the potential positive impact of physical activity initiatives on health outcomes, such as reducing chronic diseases by maintaining a healthy weight and improving the quality of life among Australian adults. This aligns with previous reviews that emphasise the broad impact of engaging in a physically active lifestyle (103, 109). Encouragingly, the study also found that some physical activity initiatives enhance social connections by encouraging participation in group activities, such as walking groups and community exercise classes, creating opportunities for social interaction (110) that may otherwise be limited, especially among vulnerable populations such as older adults, culturally diverse communities, and individuals experiencing social isolation (111).

Evaluating initiatives, strategies, and actions to increase physical activity across the population is crucial (112). However, assessing their effectiveness and impact is challenging, as many documents often lack clear indicators of efficacy or impact. In line with these findings, a recent scoping review of physical activity initiatives in rural regions revealed that seven of the 11 included studies did not report any results on physical activity or health outcomes (113), highlighting that evaluations of such initiatives are often missing. Taylor et al. (114) mentioned that the effectiveness of physical activity findings is difficult to identify due to the limited number of studies and insufficient information on population diversity, initiative methods, and outcome measures. Furthermore, most initiatives in this scoping review did not mention the type of program evaluation or assessment undertaken. Clear reporting of the evaluation framework or type used in physical activity initiatives is essential, as it ensures transparency, enhances the quality of reporting, and allows comparability across initiatives (18, 112). In addition, the limited number of published evaluations suggests restricted knowledge sharing within the public health sector, meaning that valuable insights remain localised rather than contributing to a national or global evidence base (115). This study identified that although many strategy documents were well-developed and demonstrated strong planning, their implementation was often limited, poorly executed, or not transparently communicated. This highlights a gap between the government's physical activity strategic documents and their real-world implementation. Without comprehensive reporting, it becomes challenging to assess the true impact of these programs on public health outcomes.

The sample size and participant characteristics were not consistently reported across all studies, given that the reach of these initiatives varied significantly across different demographics, methods, and presented data. The findings of this scoping review identified that physical activity initiatives across all Australian states and at the national level have engaged their targeted populations; however, the reach remains relatively low when considering the total Australian adult population. For example, a study of the 10,000 Steps program identified that over 550,000 participants engaged with the initiative over 20 years (116); while substantial, this represents a small proportion of the total Australian population of 25.4 million (117). Furthermore, although this review has identified that Australian federal and state governments are investing in a range of physical activity initiatives, more than half of Australian adults do not meet the national physical activity guidelines (9). This discrepancy raises questions regarding the generalisability of the studies' results to the wider population and the potential for widespread implementation (118). People from culturally and linguistically diverse (CALD) backgrounds are often less likely to engage in physical activity than the general population, as highlighted in previous research (119–122). This review highlighted that few initiatives specifically targeted CALD groups and other priority populations, such as First Nations individuals, migrants, and people with disabilities. This aligns with prior research, which reported limited attention to these populations (102, 114, 123). Overall, the findings of this review revealed a notable lack of physical activity initiatives targeting rural and remote areas. This gap is concerning because these regions often face unique challenges, including higher prevalence of poor health and chronic diseases and limited access to recreational facilities, healthcare services, and community resources that promote physical activity (124–127). Therefore, designers of physical activity initiatives should incorporate both traditional and digital intervention methods to effectively reach diverse groups.

### 4.2 Strengths and limitations

The main strength of this scoping review is its comprehensive examination of the existing literature on physical activity initiatives in Australia. By systematically mapping a wide range of studies and documents, this review provides valuable insights into the scope of these initiatives across different populations and settings. Including both peer-reviewed articles and grey literature enhances the breadth of the findings, capturing diverse strategies and outcomes that may not be fully represented when focusing only on peer-reviewed sources.

However, several limitations need to be acknowledged. The reliance on grey literature presented challenges, as these documents often lacked clear reporting on initiative's methodology and effectiveness, outcomes, or impact, which can limit the accuracy of the findings. While the review aimed to encompass a wide range of initiatives, the heterogeneity of the included studies may need to be clarified for direct comparisons and the generalisation of outcomes. Furthermore, this scoping review focused only on Australia, potentially limiting the applicability of its findings to broader global contexts or culturally diverse settings. While not required, this scoping review did not incorporate a quality assessment of the findings, which may have affected the strength and reliability of the conclusions. While all available avenues for gathering grey literature were thoroughly explored, it is important to acknowledge that some unpublished government documents may have remained inaccessible or undiscovered. It is possible that some organisations did not report initiatives with negative or null findings because the government has dedicated significant investment and resources to their physical activity initiatives, which may create hesitancy in sharing such finding. Therefore, this scoping review was carefully conducted with consideration of the publication bias in physical activity evaluation reports. This limitation underscores the need for ongoing efforts to identify and include all relevant data sources in future research.

## 5 Conclusion

This scoping review identifies and reports the reach, effectiveness, and impact of government-funded physical activity initiatives on physical activity and other health outcomes in Australia. Over the past 25 years, such initiatives have been implemented nationwide; however, a significant portion of the population remains unaddressed. When evaluated, these initiatives demonstrate effectiveness and yield positive impacts on health and well-being. Therefore, it is crucial for governments to increase their efforts and implement a greater number of initiatives to engage a larger proportion of the Australian population to increase population-level physical activity. While many states have published high-quality strategies and action plans, this review identified a gap in actual implementation and highlighted the need to prioritise evaluation to assess the effectiveness and impact of initiatives. Future research should focus on standardising evaluation frameworks and exploring strategies to enhance initiative sustainability and effectiveness, particularly in diverse populations. Furthermore, this review underscores the need for a more coordinated approach through a National Physical Activity Plan for Australia. While various state-based initiatives show progress, the lack of consistent implementation and evaluation frameworks across jurisdictions limits their broader impact. Therefore, we advocate for the development of a National Physical Activity Plan that not only aligns state and territory efforts but also facilitates the sharing of successful practices and lessons learned.

## Data Availability

The original contributions presented in the study are included in the article/Supplementary Material, further inquiries can be directed to the corresponding author.
