# Supplementary material for: Scoping review to assess the reach, effectiveness, and impact of government-funded, population-based physical activity initiatives in Australian adults
Source: Front Sports Act Living. 2025 Oct 10;7:1633086. doi: 10.3389/fspor.2025.1633086 (PMC12550771; doi:10.3389/fspor.2025.1633086)
Supplement: Supplementary file 2 [file Table2.docx]

**S2 Table. Search strategy and search terms**

|  | **Search Strategy** | | | | |
| --- | --- | --- | --- | --- | --- |
| **Description** | **ProQuest Public Health** | **Scopus** | **Web of Science** | **MEDLINE** | **PubMed** |
| Physical activity | ("Physical* activ*" OR physica* OR activ* OR movemen* OR exercis* OR spor* OR "active travel" OR "active transport" OR "active community" OR walk* OR jog* OR cycl* OR fitness OR "resistance training" OR recreation OR "moderate activity" OR "vigorous activity" OR "strength training") | Physical* activ*” OR physica* OR activ* OR movemen* OR exercis* OR spor* OR “active travel” OR “active transport” OR “active community” OR walk* OR jog* OR cycl* OR fitness OR “resistance training” OR recreation OR “moderate activity” OR “vigorous activity” OR “strength training” | “Physical* activ*” OR physica* OR activ* OR movemen* OR exercis* OR spor* OR “active travel” OR “active transport” OR “active community” OR walk* OR jog* OR cycl* OR fitness OR “resistance training” OR recreation OR “moderate activity” OR “vigorous activity” OR “strength training” | ((Physical* active* or physica* or activ* or movemen* or exercis* or spor* or active travel or active transport or active community or walk* or jog* or cycl* or fitness or resistance training or recreation or moderate activity or vigorous activity or strength training) and (Interventio* or involvemen* or implementatio* or applicatio* or implicatio* or initiativ* or plan* or strateg* or step* or act* or approach) and (Population or Population-based or community based or community wide or population related or broader population or adult population or adul* or over 18 or health promot*) and (Government* or cabinet or federal government* or state government* or local government* or regional government* or territory government* or Government-funded or government-funding) and (Australi* or New South Wales or Victoria or Tasmania or Queensland or Western Australia or South Australia or Australian Capital Territory or Northern Territory)). | (((("Physical* activ*" OR physica* OR activ* OR movemen* OR exercis* OR spor* OR "active travel" OR "active transport" OR "active community" OR walk* OR jogging OR cycl* OR fitness OR "resistance training" OR recreation OR "moderate activity" OR "vigorous activity" OR "strength training") AND (Interventio* OR involvemen* OR implementatio* OR applicatio* OR implicatio* OR initiativ* OR plan* OR strateg* OR step* OR action OR activity OR acts OR approach)) AND (Population OR "Population-based" OR "community based" OR "community wide" OR "population related" OR "broader population" OR "adult population" OR adul* OR "over 18" OR "health promot*")) AND (Government* OR cabinet OR "federal government*" OR "state government*" OR "local government*" OR "regional government*" OR "territory government*" OR "Government-funded" OR "government-funding")) AND (Australi* OR "New South Wales" OR Victoria OR Tasmania OR Queensland OR "Western Australia" OR "South Australia" OR "Australian Capital Territory" OR "Northern Territory"). |
| Initiative | AND (Interventio* OR involvemen* OR implementatio* OR applicatio* OR implicatio* OR initiativ* OR plan* OR strateg* OR step* OR act* OR approach) | AND Interventio* OR involvemen* OR implementatio* OR applicatio* OR implicatio* OR initiativ* OR plan* OR strateg* OR step* OR act* OR approach | AND Interventio* OR involvemen* OR implementatio* OR applicatio* OR implicatio* OR initiativ* OR plan* OR strateg* OR step* OR act* OR approach |  |  |
| Population-based | AND Population OR "Population-based" OR "community based" OR "community wide" OR "population related" OR "broader population" OR "adult population" OR adul* OR "over 18" OR ("health promoters" OR "health promoting" OR "health promotion") | AND Population OR “Population-based” OR “community based” OR “community wide” OR “population related” OR “broader population” OR “adult population” OR adul* OR “over 18” OR “health promot*” | AND Population OR “Population-based” OR “community based” OR “community wide” OR “population related” OR “broader population” OR “adult population” OR adul* OR “over 18” OR “health promot*” |  |  |
| Government funded | AND Government* OR cabinet OR ("federal government" OR "federal governmental" OR "federal governments") OR ("state government" OR "state governmental" OR "state governments") OR ("local government" OR "local governmental" OR "local governments") OR ("regional government" OR "regional governmental" OR "regional governments") OR ("territory government" OR "territory governments") OR "Government-funded" OR "government-funding" | AND Government* OR cabinet OR “federal government*” OR “state government*” OR “local government*” OR “regional government*” OR “territory government*” OR “Government-funded” OR “government-funding” | AND Government* OR cabinet OR “federal government*” OR “state government*” OR “local government*” OR “regional government*” OR “territory government*” OR “Government-funded” OR “government-funding” |  |  |
| Australia | AND Australi* OR "New South Wales" OR Victoria OR Tasmania OR Queensland OR "Western Australia" OR "South Australia" OR "Australian Capital Territory" OR "Northern Territory" | AND Australi* OR “New South Wales” OR Victoria OR Tasmania OR Queensland OR “Western Australia” OR “South Australia” OR “Australian Capital Territory” OR “Northern Territory” | AND Australi* OR “New South Wales” OR Victoria OR Tasmania OR Queensland OR “Western Australia” OR “South Australia” OR “Australian Capital Territory” OR “Northern Territory” |  |  |
| Additional limits | Year: 2000-01-01 to 2024-04-02 Document type: Article OR Report OR Case Study OR Review OR Statistics/Data Report OR Literature Review OR Government Document  County: Australia Language: English Subject: Human, Adult | Year – 2000 to 2023  Subject Area – Health Profession Document type – Article, Review, Conference Paper  Language - English  Country – Australia | Publication Date: 1/1/200 to 22/03/2023  Refined by Language: English,  Not Document Type-Meeting Abstract or letters, Book chapters, Countries/Regions: Australia, Web of Science Categories: Public Environmental Occupational Health or Multidisciplinary Sciences or Environmental Sciences or Hospitality Leisure Sport Tourism or Sport Sciences or Health Policy Services or Physiology or Behavioural Sciences or Family Studies or Primary Health Care. | English language, humans, year 2000-current, all adult (19 plus years) and (evaluation study or government publication or journal article) | To consider – Jog* =jogging  Act* =action OR activity OR act  Species-Humen  Article Language - English  Age - Age Adult: 19+ years  Young Adult: 19-24 years  Adult: 19-44 years  Middle Aged + Aged: 45+ years  Middle Aged: 45-64 years  Aged: 65+ years  80 and over: 80+ years |
