# Supplementary material for: Scoping review to assess the reach, effectiveness, and impact of government-funded, population-based physical activity initiatives in Australian adults
Source: Front Sports Act Living. 2025 Oct 10;7:1633086. doi: 10.3389/fspor.2025.1633086 (PMC12550771; doi:10.3389/fspor.2025.1633086)
Supplement: Supplementary file 3 [file Table3.docx]

**S3 Table - Characteristics of government funded physical activity initiatives - Peer reviewed documents**

| **Reference** | **Author and year** | **Jurisdiction** | **Physical activity initiative** | **Sub - Study** | **Summary of the Program** | **Research Aim/Purpose** |
| --- | --- | --- | --- | --- | --- | --- |
| (31) | Duncan et al. 2021 | National | 10,000 Steps Program | Impact of COVID-19 | 10,000 Steps is a multi-strategy physical activity promotion project that aims to raise awareness and participation in physical activity by accumulating incidental activity throughout the day.  In October 2009, the 10,000 Steps resources and programs available for workplaces were expanded to include a Workplace Guide (promoting physical activity and implementing 10,000 Steps). The program was initially aimed at office workers to combat sedentary behaviour and increase daily physical activity. | To investigate changes in physical activity reported through the 10,000 Steps program and changes in engagement with the program during the COVID-19 pandemic. |
| (32) | Vandelanotte et al. 2020 | National |  | Every Step Counts |  | To increase individual awareness of physical activity levels. To increase overall physical activity levels. To create awareness of the health benefits |
| (33) | Kirwan et al. 2012 | National |  | Smartphone Technology |  | To measure the effectiveness of a smartphone application, the iStepLog, to improve health behaviours in existing members of an online physical activity program (10,000 Steps, Australia). |
| (34) | Davies et al. 2012 | National |  | Publicly Available Physical Activity Website |  | To examine associations between exposure to Initiative components and website engagement in a publicly accessible physical activity website (www.10000steps.org.au). |
| (35) | Guertler et al. 2015 | National |  | Engagement and Nonusage Attrition |  | To examine engagement with a web-based physical activity Initiative in real life because findings from controlled settings may not translate into real-life settings. |
| (42) | Rayward et al. 2019 | Queensland |  | Free Web- and App-Based Physical Activity Initiative |  | To determine the reach of this 10,000 Steps social media campaign, assess new member registration rates as a consequence of the social media campaign and compare program engagement and time to non-usage attrition of the new members resulting from the social media campaign with 10,000 Steps users attracted using different methods |
| (43) | Duncan et al. 2018 | Queensland |  | A community-wide eHealth physical activity promotion program |  | Goal setting (10,000 steps per day) and self-monitoring (the use of a pedometer to track daily step counts) were fundamental aspects of this program. |
| (44) | Cristina et al. 2018 | Queensland |  | Workplace-Based Microgrants to Improve Physical Activity |  | To examine the feasibility and acceptability of the 10,000 Steps Pedometer Microgrant Scheme by exploring the perceptions and opinions of key employee representatives who applied for and received a 10,000 Steps Pedometer Microgrant for their workplace. |
| (36) | Rissel and Watkins 2014 | National | National cycling skills program (AustCycle) in Australia 2010-2013 | N/A | From 2010 to 2013 AustCycle implemented a community-based  national adult cycle training program across Australia funded by federal, state and local governments, workplaces and individuals.  A primary aim for Commonwealth Health Department funding was reduction of risk factors for chronic disease. | To assess the impact on cycling behaviour (including skills and confidence) of the AustCycle program, and its impact on weight of participants. |
| (37) | O’Callaghan et al. 2022 | New South Wales | Get Healthy Information and Coaching Service® (GHS) | Initiative for Chinese community | In 2015 the GHS developed a pilot program for Chinese (Mandarin and Cantonese-speaking) communities using bilingual coaches and translated material to address these barriers. | To address the health needs of the Chinese people in a culturally and linguistically appropriate way including the acceptance of the concept of coaching by participants**.** |
| (39) | Fjeldsoe et al. 2014 | New South Wales |  | Protocol of evaluation | The Get Healthy Information and Coaching Service® (GHS) is a free, publicly available, telephone-delivered coaching program targeting healthy lifestyle improvements (moderate weight loss, physical activity and dietary behaviours) in adults. | To evaluate the feasibility, acceptability and efficacy of a text message-delivered extended contact Initiative to enhance or maintain change in physical activity, dietary behaviour and weight loss among participants who have completed a six-month Government-funded, population-based telephone coaching lifestyle program: the Get Healthy Information and Coaching Service (GHS) |
| (40) | Crane et al. 2019 | New South Wales | Get Healthy at Work (GHaW), a state-wide workplace health promotion program in Australia | N/A | GHaW is a comprehensive workplace health promotion program, developed under the Healthy Workers Initiative. GHaW aims to encourage businesses to participate in preventing lifestyle-related chronic diseases among workers. | To evaluate the state-wide implementation of a complex WHP in Australia and to assess its short-term impacts at the business level. |
| (41) | McGill et al. 2023 | New South Wales | Get Healthy in Pregnancy (GHiP) Program | N/A | The GHiP program is a free telephone coaching program, aimed to encourage healthy eating and active living behaviours, that is available to pregnant women aged 16+ years in New South Wales, Australia. GHiP is delivered by university-qualified coaches through a program provider. The program was first offered in July 2015 to achieve healthy GWG in line with the IOM (Institute of Medicine) guidelines. | To encourage healthy eating and active living behaviours among pregnant women aged 16+ years in New South Wales, Australia.  To investigate the effectiveness of the GHiP program as it is implemented at scale. |
| (38) | Hetherington et al. 2015 | National | Healthy Eating Activity and Lifestyle (HEAL™) program | N/A | The HEAL™ program is a lifestyle modification program that enables participants to develop lifelong healthy eating and physical activity behaviours. The HEAL program is an eight-week, group-based lifestyle modification program designed for people with, or at risk of developing, chronic conditions. | To report on the effectiveness of the Healthy Eating Activity and Lifestyle (HEAL) program, a program funded under the Australian government’s Healthy Communities Initiative. |
| (45) | Clarke et al. 2020 | Victoria | LiveLighter® Obesity prevention program | Obesity prevention policy processes | The LiveLighter® program is a public health education campaign  aimed at encouraging Australians to lead healthier lifestyles. It focuses on helping people eat well, be physically active, and avoid excess weight gain, which can reduce the risk of chronic diseases. | To examine the influences on the decision by the Victorian Government to fund the LiveLighter® campaign, using multiple theories of the policy process to underpin the analysis. |
| (46) | Miller et al. 2022 |  |  | LiveLighter(®) campaign |  | To test whether similar improvements in campaign awareness; knowledge of key messages; perceived effectiveness; and changes in physical activity and dietary intentions could be achieved in another state, albeit, with a reduced media buy. |
| (47) | Thomas et al. 2009 | Victoria | The VicHealth MetroACTIVE Demonstration Grants Program | N/A | The Metro ACTIVE Program is intended to influence local  governments to adopt an integrated planning approach to support residents to be physically active. | To strengthen local government capacity to adopt integrated planning to promote physical activity. |
| (48) | Lyle et al. 2008 | New South Wales | The WellingTonne Challenge | N/A | The WellingTonne Challenge was a whole-of-community project designed to support a small rural community to lose weight and reduce their risk of chronic disease. It was devised by staff from the Wellington Community Health Centre in response to a realisation that many people accessing the Centre’s services sought advice on weight loss, and that clients often  accessed different chronic disease prevention services  which could be better coordinated. | To mobilise the community and support overweight or obese residents to lose weight |
| (49) | Byrne et al. 2019 | Tasmania - Launceston | A community-wide physical activity program in Launceston, Australia | N/A | Active Launceston adopted a service-oriented population-based approach with a goal to mobilise community members to increase their participation in physical activity by filling gaps in provision, reducing barriers and targeting those with the highest need. | To improve health and well-being through physical activity |
| (50) | Caperchoine et.al 2009 | New South Wales, Victoria, Australian Capital Territory, and Queensland | Women’s Active Living Kits (WALK) Pilot Program | N/A | The WALK Pilot Program was an Australian federal government initiative designed to identify an effective model for extending physical activity participation in government identified priority women’s groups. | To address the barriers and challenges to physical activity participation in selected women’s groups and present possible strategies to assist with engaging these groups in physical activity. |
| (51) | Jose et al. 2022 | Tasmania | Trips4health | The COVID-19 disrupted trips4health | Partnering with a public transport provider, state government, and local government, the single-blinded randomised controlled trial, trips4health, investigated the impact of public transport use incentives on transport-related physical activity in Tasmania, Australia | To conduct a process evaluation of the COVID-19 disrupted trips4health study. |
| (52) | Evans et al. 2023 |  |  | Adults’ public transport use for physical activity gain. |  | To determine the impact of a financial incentive-based strategy on transport-related and total physical activity via increased public transport use. |
|  |  |  |  |  |  |  |
