# Supplementary material for: Scoping review to assess the reach, effectiveness, and impact of government-funded, population-based physical activity initiatives in Australian adults
Source: Front Sports Act Living. 2025 Oct 10;7:1633086. doi: 10.3389/fspor.2025.1633086 (PMC12550771; doi:10.3389/fspor.2025.1633086)
Supplement: Supplementary file 4 [file Table4.docx]

**S4 Table - Characteristics of the government funded physical activity Initiatives - Peer reviewed documents**

| **Study ID** | **Physical activity initiative** | **Sub - Study** | **Physical activity type** | **Intervention Type** | **Evaluation Type** | **Duration and year of the Initiative** | **Population and sample size within the source of evidence** | **Study Design** |
| --- | --- | --- | --- | --- | --- | --- | --- | --- |
| (31) | 10,000 Steps Program | Impact of COVID-19 | Walking | Web- and mobile-based physical activity promotion | Not reported | Two years in 2018-2020 | 2,995,617 days (84.4%) of data between January 1, 2018, and June 30, 2020, were included in the analysis | Prospective Study |
| (32) |  | Every Step Counts |  | e- and mHealth Initiative | Ongoing evaluation (Proposed) | Since 2009 | n=425,000 | Not reported |
| (33) |  | Smartphone Technology |  | Developed iStepLog application to monitor daily step count | Not reported | Three months in 2009 (August to October) | n = 50 (Initiative group)  n=150 (Control group) | A 2-arm matched case–control trial |
| (34) |  | Publicly Available Physical Activity Website |  | Website-delivered physical activity Initiatives | Not reported | Two years in 2006-2008 | n= 348 | A prospective study |
| (35) |  | Engagement and Nonusage Attrition |  | Web-based physical activity Initiative | Not reported | Eight months in 2013-2014 | n=17,590 | Quantitative research design |
| (42) |  | Free Web- and App-Based Physical Activity Initiative |  | Social media campaign | Not reported | Six Weeks in 2018 | n=1242 | Quantitative research design |
| (43) |  | A community-wide eHealth physical activity promotion program |  | Ongoing website development with resources to support implementation | Ongoing evaluation (Proposed) | Six Months in 2016 | 200 workplaces | Not reported |
| (44) |  | Workplace-Based Microgrants to Improve Physical Activity |  | The Pedometer Microgrant Scheme | Not reported | One month Initiative (August and October 2014 and June and July 2015) | n = 19 (Interview participants) | An exploratory study |
| (36) | National cycling skills program (AustCycle) in Australia 2010-2013 | N/A | Cycling | A system of cycle training. | Not reported | Three years in 2010 – 2013 | n=4145 | Cross sectional survey design |
| (37) |  | Initiative for Chinese community |  | Bilingual/bicultural personalized coaches Translation of the written resources | Not reported | Four years in 2015-2019 | n=35 | A Qualitative study design |
| (39) | Get Healthy Information and Coaching Service® (GHS) | Protocol of evaluation | Walking and physical activity | Telephone coaching | Maintenance evaluation | Six months in 2014 | n=212 | A randomised controlled trial |
| (40) | Get Healthy at Work, a state-wide workplace health promotion program in Australia | N/A | Physical activity | Health education and action plans | Impact Evaluation | One year in 2014 | Baseline (n=244) 6 month (n=94) 12 months (n=56) | A mixed-method design |
| (41) | Get Healthy in Pregnancy Program | N/A | Walking and vigorous physical activity | Telephone coaching | Impact Evaluation | One year in 2018-2019 | n=3702 | Retrospective analysis of routinely collected data |
| (38) | Healthy Eating Activity and Lifestyle (HEAL (TM)) program | N/A | Physical activity | Education sessions | Not reported | Eight weeks in 2010-2013 | n = 2827 | Mixed method design |
| (45) | LiveLighter ® Obesity prevention program | Obesity prevention policy processes | Physical activity | Policy Initiative | Not reported | N/A | n=11 stakeholders | Qualitative design |
| (46) |  | LiveLighter(®) campaign |  | Social marketing campaign | Not reported | Six Weeks in 2014 | 1000 Survey participants from each state (Baseline data of Vic and SA)  1502 for follow up survey participants (Vic: n = 715; SA: n = 787) | A controlled cohort design |
| (47) | The VicHealth MetroACTIVE Demonstration Grants Program | N/A | Physical activity | Integrated planning approach | Impact Evaluation | Two years in 2005-2007 | n=117 | A qualitative mixed-method approach |
| (48) | The WellingTonne Challenge | N/A | Walking | Multiple Initiatives  Community engagement  Weight loss challenge  Behavioural change  Support and resources | Impact evaluation | 12 Weeks (Implementation year is not reported) | n= 371 | Scales measurements and the questionnaire |
| (49) | A community-wide physical activity program in Launceston, Australia | N/A | Walking, running, cycling, dancing, hydrotherapy, archery, orienteering, 18yoga, tai chi, rock climbing, sailing and laser tag | Free community-based physical activity programs and events | Process Evaluation and Impact Evaluation | Seven years in 2008-2015 | n=11,887 | A mixed-methods research design |
| (50) | Women’s Active Living Kits (WALK) Pilot Program | N/A | Physical activity | Evaluation Sessions | Not reported | One year in 2005-2006 | n=78 | Qualitative Design |
| (51) | Trips4health | The COVID-19 disrupted trips4health | Transport related physical  activity | Incentive-based strategies | Process Evaluation | Six weeks in 2019-2020 | n=116 (participants) n=11 (Initiative participants, and PT provider staff interviewed) | A single-blinded randomised controlled trial |
| (52) |  | Adults’ public transport use for physical activity gain. |  | Incentive-based strategies | Not reported |  | N=110 | A single-blinded randomised controlled trial |
