# Supplementary material for: Scoping review to assess the reach, effectiveness, and impact of government-funded, population-based physical activity initiatives in Australian adults
Source: Front Sports Act Living. 2025 Oct 10;7:1633086. doi: 10.3389/fspor.2025.1633086 (PMC12550771; doi:10.3389/fspor.2025.1633086)
Supplement: Supplementary file 5 [file Table5.docx]

**S5 Table – Summary of the Reach, Effectiveness, and Impact of the Population Based Government Funded Physical Activity Initiatives – Peer Reviewed documents**

| **Study reference** | **Physical Activity Initiative** | **Sub - Study** | **Reach** | **Effectiveness** | **Overall Effectiveness  [Positive (+), Negative (-), Neutral (0), Not Applicable (N/A)]** | **Impact** |
| --- | --- | --- | --- | --- | --- | --- |
| (31) | 10,000 Steps Program | Impact of COVID-19 | 60,560 members were logged step data for at least 7 days. | During the lockdown, the use of the program increased steeply.  - More than 450 new users and more than 15 new organizations registered per day.  - On the peak day, there were more than 9000 step entries per day, with nearly 100 million steps logged per day.  - On average per day, there were about 55 new registered users (*P*<.001), 2 new organizations (*P*<.001), 25.6 million steps (*P*<.001), and 2672 log entries (*P*<.001) more in 2020 compared to the same period in 2019.  The pandemic led to a significant reduction in physical activity among 10,000 Steps members. The reduction was observed for average steps of 7 days (3.4%; P<.001) and 30 days (5.0%; P<.001) before and after the lockdown.  New South Wales, Australian Capital Territory, and Victoria had the greatest step reductions, with decreases of 7.0% (P<.001), 6.2% (P=.02), and 4.7% (P<.001), respectively. | +  - | Study results relived that the event with the biggest negative impact on steps was the lockdown starting at the end of March 2020. Lockdowns, social distancing measures, and restrictions on movement contributed to a decline in daily step counts. |
| (32) |  | Every Step Counts | To date the project has signed-up over 425,000 members who have logged 221 billion steps (~43 million a day) on the website or app. | In 2011, the Queensland Government designated the 10,000 Steps program as their key physical activity workplace health promotion strategy.  10,000 Steps is included in the Australian Department of Health’s Obesity Prevention Program's resource materials.  10,000 steps included in the Implementation Plan for the Healthy Workers' Initiative of the Australian Government’s National Partnership Agreement on Preventive Health 2010-2013.  Awareness of 10,000 Steps in the Australian  population has consistently been found to be much higher than the awareness of other Australian health promotion programs.  Workplace Challenge' has been used by ∼65% of 10,000 Steps members, which on average increases physical activity by 159 min/week for those who participate in it. | + | The 10,000 Steps program has had a considerable impact on increasing physical activity levels, improving health outcomes, and fostering community engagement. By setting a clear, manageable goal and providing tools for tracking and motivation, the program effectively encourages individuals to integrate more physical activity into their daily lives, with broad implications for personal health and public health outcomes. |
| (33) |  | Smartphone Technology | Initiative participants used the application 71.22% (2210/3103) of the time to log their steps. | Use of the application was associated with an increased likelihood to log steps daily during the initiative period (average 62 days) compared with (41 days) those not using the application  Linear regression analysis revealed a nonsignificant relationship between perceived usability (r = 0.216, P = 0.21) and usefulness (r = 0.229, P = 0.17) of the application and frequency of logging steps in the Initiative group. | +  0 | The impact of this study lies in its potential to demonstrate whether smartphone technology can effectively support and enhance participation in physical activity programs like the 10,000 Steps Program, leading to improved health outcomes and more efficient program design. |
| (34) |  | Publicly Available Physical Activity Website | The study involved 348 members of the 10,000 Steps program who completed a web-based survey and had their engagement tracked over two years | Overall engagement with the 10,000 Steps website was high, and the results demonstrate the relative effectiveness. | + | Health-related websites and online physical activity Initiatives help increase physical activity participation engagement among population. |
| (35) |  | Engagement and Nonusage Attrition | The study analysed data from 16,948 users | The 10,000 Steps program demonstrated higher engagement compared to other freely accessible Web-based health behaviour Initiatives highlighting the effectiveness of incorporating a smartphone app to enhance user engagement and reduce attrition compared to Web-only users.  In the subsample, 50.00% (5826/11,651) of users stopped logging physical activity through the program after 30 days.  Personal factors associated with reduced nonusage attrition risk were being male, non-Australian, and older age. | +  -  + | Using smartphone apps enhances physical activity program engagement and lowers attrition among the population. |
| (42) |  | Free Web- and App-Based Physical Activity Initiative | A total of 1242 new Queensland users registered with the 10,000 Steps program during the social  media campaign period. | A statistically significantly higher number of daily new Queensland users of the 10,000 Steps program registered during the social media campaign period compared with both the six weeks prior to the social media campaign and the same time one year prior to the social media campaign. The social media campaign was effective in promoting awareness of the 10,000 Steps program. | + | Risk of non-usage attrition was higher among new users from the Socia media campaign than new users from one year prior. |
| (43) |  | A community-wide eHealth physical activity promotion program | Between July and December 2016, 200 workplace challenges were started. They included 2004 teams with 13 920 members. | Participation in the Workplace Challenge significantly increased the proportion of employees who report sufficient physical activity  The form of a ‘virtual journey’ facilitated the reaching of individuals at walking and activity levels. | N/A | The Workplace Challenge helps to engage who are at higher risk of chronic disease and motivated them to be active. |
| (44) |  | Workplace-Based Microgrants to Improve Physical Activity | All 34 Queensland-based organizations who were awarded a Pedometer Microgrant in round two were purposively invited to participate. Also 1575 pedometers were provided to the organisations. | The study found that the microgrant scheme was effective in promoting physical activity among employees. | + | The microgrant scheme demonstrated potential for long-term sustainability of workplace health promotion activities.  Pedometer Microgrant Scheme impact on increase physical activity awareness and of employees. In addition, it was an initial Initiative to discussed about other health topic such as smoking and healthy eating.  This program helped reduce absenteeism and presenteeism, lowered medical costs, and improved productivity, morale, and job satisfaction. |
| (36) | National cycling skills program (AustCycle) in Australia 2010-2013 | N/A | 4145 participants were registered to the program. | The effectiveness of the Initiative is demonstrated by the follow-up surveys. At the three-month mark, 74.5% of participants engaged in at least 150 minutes of physical activity per week. By the 12-month follow-up, the average weekly physical activity time increased to 431.5 minutes, with 90.7% of respondents meeting the recommended levels of physical activity. This indicates significant and sustained improvements in physical activity levels among participants. | + | At baseline, the overall mean body mass index (BMI) was 26.2, with 48.9% of participants overweight or obese. For participants with both baseline and three-month follow-up weight data (*n*=338), there was a mean reduction in BMI of 0.24 which is a statistically significant reduction (*p*=0.006) and representing an average.72 kg weight loss. Overall, 52.3% of participants reduced their weight at follow-up, and 28% had increased it. For participants with both baseline and 12-month follow-up weight data (*n*=102), there was a mean reduction in BMI of 0.52, also statistically significant (*p*=0.025), representing an average 1.58 kg weight loss. |
| (37) | Get Healthy Information and Coaching Service® (GHS) | Initiative for Chinese community | At the end of the pilot CGHS program, 162 people enrolled in the GHS, with 26 selecting information-only and 136 receiving bilingual coaching on the phone. | Stakeholders and participants felt that the CGHSH program linguistically and culturally promoted physical activity. | **+** | GHS in Australian Chinese communities has been the improvement of health outcomes (improve their chronic health conditions after six months of Initiative.) through better access to culturally and linguistically appropriate services.  Research participant agreed that the CGHS was relevant and addressed the communities' health needs, especially for older people  There might be challenges in ensuring participants' sustained motivation and goal achievement. |
| (39) |  | Protocol of evaluation | Aged over 18 adult population (Expected) | This program expected to increase population engagement for the physical activity. | N/A | GHS randomized controlled trial protocol evaluation expected enhance or maintain the long-term behaviour change in physical activity, dietary and weight loss among participants.  This program expected behaviour change in maintaining body weight and weight loss among the NSW population during the GHS implementation period. |
| (40) | Get Healthy at Work, a state-wide workplace health promotion program in Australia | N/A | In 2014, 486 businesses registered for the GHaW program. | Not reported | N/A | Employees were generally healthy (p = 0.045-time X group effect) and that the workplace promoted healthy behaviours (p = 0.004 time X group effect) improved significantly.  48.4% (n=31) Businesses participating in GHaW reported positive changes in workplace culture over time. Employees perceived their workplaces as promoting healthier behaviours  While there were improvements in health perceptions and workplace culture, changes in perceptions about work productivity were significantly not observed |
| (41) | Get Healthy in Pregnancy Program | N/A | Of the 3702 women enrolled in the GHiP program throughout the evaluation period. | More pregnant women enrolled during 2019 (63.8%) than 2018 (36.1%)  After participating in the GHiP program,  significant improvements were noted in relation to the proportion of women meeting the guidelines (from 39.7% to 50.5%) for physical activity  Women who graduated from the program that reported positive health-related behaviour changes in increased sessions of walking (0.8 session/week, p < 0.001), vigorous physical activity (0.3 sessions/week, p < 0.001) | + | A higher proportion of women with pre-pregnancy obesity gained weight below the guidelines compared to those who gained above the guidelines |
| (38) | Healthy Eating Activity and Lifestyle (HEAL (TM)) program | N/A | 2827 participants had started in a HEAL program and 61% of participants had completed the program and returned for post-program testing. | At baseline, 60% of participants were not meeting recommended physical activity levels (210 min per week) while at post-program testing this had dropped to 45%  HEAL participants were found to have significantly increased the quantity and frequency of their weekly physical activity and reduced their average daily sitting time (p<0.001). | + | HEAL Program has involved to participants’ behaviour changed for physical activity engagement and other health metrics. |
| (45) | LiveLighter ® Obesity prevention program | Obesity prevention policy processes | The LiveLighter® campaign was designed to target a broad audience across Victoria, Australia. | By employing political science and systems thinking, the study explores how physical activity is integrated into the campaign, evaluates the effectiveness of these efforts, and provides insights for improving the promotion of physical activity as a key component of obesity prevention strategies | N/A | The study highlighted several key influences on policy decision-making, including external events, evidence of the problem, Initiative effectiveness, and resistance from various stakeholders. |
| (46) |  | LiveLighter(®) campaign | The campaign was funded for two media waves, achieving a total of 1695 target audience rating points (TARPs), indicating significant reach and frequency | The study result indicated that there was no significant relationship between baseline data of Victoria and South Australia for Campaign outcomes for Physical activity intention (Being more active, sitting less and walking more) - p>0.005 | 0 | Campaign awareness was moderate, with 61.5% of respondents being aware of the campaign.  Perception was high, as 91.0% found it believable, 87.3% saw it as a strong argument for reducing weight, and 55.6% considered it relevant. In terms of behavioural impact, the campaign motivated 59.1% of those exposed to take action toward achieving a healthy weight. |
| (47) | The VicHealth MetroACTIVE Demonstration Grants Program | N/A | The VicHealth MetroACTIVE Demonstration Grants Program provided funding to six Victorian metropolitan local governments to conduct 2-year projects to support the use of integrated planning for physical activity. | Three out of six metropolitan councils in Melbourne showed evidence of integrated planning for physical activity. The remaining councils focused more on delivering community participation programs. | N/A | Integrated planning is viable for developing a coordinated strategy to promote physical activity across various council services and functions. Ongoing strategy, senior management commitment, and organizational capacity also help integrate planning for physical activity Initiatives.  Successful integrated planning was linked to leadership from senior management and an organizational culture that supported collaboration across council departments.  Barriers included complex organizational structures, high demands due to growing residential populations, and a poor climate among staff. |
| (48) | The WellingTonne Challenge | N/A | A total of 371 eligible participants registered for the WellingTonne Challenge. | Based on self-reported behaviour change the proportion of people walking for exercise in the previous week had increased by 16%, and those achieving a level associated with health benefit more than doubled to 34% after 12 weeks. | + | Participants achieved an average weight loss of around 3 kg each.  Active participants collectively lost 687 kg by the end of the 12-week program, which was below the goal of 1000 kg.  The initiative demonstrated the potential for community-based Initiatives to make a meaningful impact on public health in rural areas. |
| (49) | A community-wide physical activity program in Launceston, Australia | N/A | Active Launceston attracted 11,887 attendees during the implementation period. | There was a significant increase in vigorous physical activity in 2012 and 2015 compared to 2008.  A higher proportion of participants achieved sufficient activity for health in 2015 compared to 2008  There was no significant difference in the proportion of people walking in 2012 and 2015 compared to 2008  While the overall proportion of people participating in any physical activity remained similar across the years (2008: 77.7%, 2012: 77.1%, 2015: 73.6%). | +  0 | Focus groups highlighted benefits such as increased engagement in exercise, better health, and enhanced social connectedness |
| (50) | Women’s Active Living Kits (WALK) Pilot Program | N/A | Overall, 78 women participated in 10 focus group sessions about the WALK program. | Participants highlighted strategies they felt would enable physical activity participation. | N/A | Participants reported a number of psychological and cognitive, sociocultural, and environmental factors which restricted their participation in physical activity. |
| (51) | Trips4health | The COVID-19 disrupted trips4health | 116 participants completed baseline measures, with 110 randomized and 64 completing post-Initiative measures. | Due to early cessation of trips4health because of COVID-19, data were unavailable on the longer-term impact of financial incentives on bus use and PA. | N/A | 90% of participants found bus trip incentives helpful, and 59% reported increased motivation to use Public Trasport (PT)  59% reporting the incentives motivated them to use PT more |
| (52) |  | Adults’ public transport use for physical activity gain. | The study involved 110 Australian adults who were infrequent bus users (using the bus ≤2 times/week) | The Initiative group saw an increase in transport-related physical activity by 80.0 minutes/week (133.3%) and 264.0 MET-minutes/week (133.3%).  The Initiative group, who received weekly motivational texts and bus credits, showed a smaller decline in steps per day compared to the control group during the COVID-19 pandemic. Specifically, the Initiative group had a decrease of -557.9 steps (-7.9%) vs.-1018.3 steps/week (-13.8%). | +  - | This study suggested that financial incentives can effectively increase public transport use and related physical activity. Due to the COVID-19 pandemic, this program could not achieve the long-term impacts of the program. |
