# Supplementary material for: Scoping review to assess the reach, effectiveness, and impact of government-funded, population-based physical activity initiatives in Australian adults
Source: Front Sports Act Living. 2025 Oct 10;7:1633086. doi: 10.3389/fspor.2025.1633086 (PMC12550771; doi:10.3389/fspor.2025.1633086)
Supplement: Supplementary file 6 [file Table6.docx]

**S6 Table – Characteristics of Grey Literature Evaluation Reports**

| **Study Reference** | **Physical Activity Initiative** | **Reports’ Version** | **Summary of the Programme** | **Jurisdiction, Program Starting year and End year** | **Physical Activity Type** | **Intervention Type** | **Evaluation Type** | **Documentation Type** |
| --- | --- | --- | --- | --- | --- | --- | --- | --- |
| (53) | Heart Foundation Walking | Heart Foundation Walking. Active Body Active Brain pilot | Heart Foundation Walking is Australia’s largest free walking network. Since 1995, the Heart Foundation has offered a Walking program to support people in being more active and aware of the risk factors for cardiovascular and other chronic diseases. The Walking Program aims to encourage more people to be more active more often.  Active Brain pilot Heart Foundation Walking has joined in partnership with ACH Group, a leading provider of specialist services for older people and those with dementia and received funding from Alzheimer’s Australia’s National Quality Dementia Care Initiative. The goal of the project was to improve access to, and the uptake of, HFW groups for people with dementia and their careers. | South Australia, 2012-2013 | Walking | Walking Sessions | Post-Quality of Life – Alzheimer’s Disease (QOL-AD) evaluation tool. | Summary report |
| (54) |  | Heart Foundation Walking 2016 report |  | National, 1995 - Continuing |  | Walking campaign through media releases, electronic direct marketing and social media,  Host Organisations and nominated Local  Coordinator/s to set up walking groups in their community or workplace. | Not specified | Evaluation Report |
| (55) |  | Heart Foundation Walking 2018 report |  |  |  | Host Organisations and nominated Local  Coordinator/s to set up walking groups in their  community or workplace, Awareness Programs, Media Campaigns (TV commercials, radio releases and social media posts) | Not reported | Annual Review |
| (56) | Queensland Walking Strategy Progression Report 2019-2021 | N/A | The Queensland Walking Strategy Progress Report 2019-2021 provides insights into the state’s efforts to make walking an easy choice for everyone. The Action Plan for Walking 2019-2021 included 44 actions – 35 are completed and nine are ongoing. This report highlights some of the achievements. | Queensland, 2019-2021 | Walking | Strategy and actions | Not reported | Progression Report |
| (57)  (59)  (58) | Queensland state of cycling report 2019  Queensland state of cycling report 2022  Queensland Cycling Action Plan 2020–2022 - Progression Report 2021 | 2017-2019 report  2020-2021 report  2020-2022 progression report | The Queensland State of Cycling Reports provides key data on bike riding in Queensland and tracks progress under the objectives of the Queensland Cycling Strategy 2017–2027. The Queensland State of Cycling Report tracks the Queensland Government’s progress towards achieving the vision of ‘more cycling, more often’  In September 2020, the Queensland Government released the Queensland Cycling Action Plan 2020–2022. | Queensland, 2017-2019,  2020-2021,  2022-2022 | Cycling | Strategy and actions | Not reported | Progression Report |
|  |  |  |  |  |  |  |  |  |
| (87) | NSW Get Healthy Information and Coaching Service | 2009 Report | The NSW Get Healthy Information and Coaching Service®(GHS), a telephone-based information and coaching service about healthy eating, being active, and achieving and maintaining a healthy weight for NSW adults. A mass media campaign, consisting mainly of television advertising, was launched in March 2009 to promote the NSW GHS.  In July 2010, GHS was launched in Tasmania as one of several Intervention to combat chronic disease. High rates of chronic disease in Tasmania are having an adverse impact on the state healthcare system and economy. The recruitment is based on two ways:  Self-referral: Mass media and local promotions,  Secondary and other referral: GP and other health care providers’ referral and direct marketing to targeted households that includes a letter of introduction to the Service. | New South Wales, 2009 – Continuing | Physical activity and Walking | A mass media campaign, - Television, radio and print advertising. - GHS-specific and re-badged National ‘Measure Up’ campaign television advertisement | Process Evaluation | A paper/ A brief report |
| (67) |  | 2009-2013 Report |  |  |  | Information only: GHS Participants seeking ‘information only’ receive an evidence-based printed information package on healthy eating, physical activity and achieving and maintaining a healthy weight.  Six-month coaching program,  GHS Aboriginal program: In November 2012, an enhancement to the GHS for Aboriginal people commenced.  Type 2 Diabetes Prevention Module: In July 2013, the GHS launched a type 2 Diabetes Prevention Module to address the high burden of disease from this chronic disease. |  | Evaluation Report |
| (68) | The story of the Tasmanian Get Healthy Information & Coaching Service | 2009 – 2013 and GHS Tasmania |  | Tasmania, 2010 - Continuing |  | Information only  Six-month coaching program, |  |  |
| (69) | This Girl Can – Victoria 2018 and 2021 | 2018 Report | This Girl Can—Victoria launched in 2018 with the aim of motivating and empowering women to be active. This program focuses on increasing physical activity among Victorian women, with a focus on less active women. It also supports gender equality by challenging traditional gender roles and stereotypes in sports and celebrating women in this space. The campaign focuses on women aged 18 and over who are less active. | Victoria, 2017 - 2023  Sports and physical activity | | Comprehensive social marketing approach  State-wide mass media advertising,   Stakeholder engagement,   Funded sports partnerships. | Process Evaluation | Campaign report |
| (70) |  | 2021 Report |  |  |  |  |  |  |
| (71) | Be Active program evaluation highlights Improving physical activity outcomes in local communities | N/A | The Be Active program aimed to increase physical activity participation in children, older people and families, and improve civic engagement through volunteerism. Be Active ran in tandem with the Victorian Government’s Healthy Together Victoria initiative, to boost local governments’  efforts to achieve increased physical activity participation in their communities. | Victoria, 2012-2015 | Physical activity | Strengthening local policy and planning, including Municipal Public Health and Wellbeing Plan  The delivery of innovative strategies to increase participation in physical activity and civic engagement through volunteering within local communities  Active travel, walking and cycling initiatives  Workplace initiatives  Initiatives focused on children, families and older people. | Evaluation - Not specified | Evaluation Highlights |
| (85) | 10,000 Steps Workplaces Evaluation | N/A | In October 2009, the 10,000 Steps resources and programs available for workplaces were expanded to include a Workplace Guide (promoting physical activity and implementing 10,000 Steps), additional promotional materials and additional workplace resources to aid the promotion of physical activity in the workplace. The program was initially aimed at office workers to combat sedentary behaviour and increase daily physical activity. | National, 2009 - Continuing | Walking | Workplace-based physical activity and 10000 Steps Initiative. | National Evaluation | Evaluation Report |
| (86) | Community Activation Program | N/A | The Community Activation Program was an innovative VicHealth initiative that aimed to assist less-active people in becoming more active. This program connected five Victorian councils to create and ‘activate’ under-used public spaces within local communities, increasing access to opportunities for physical activity and social connection. | Victoria, 2015 -2016 | Physical Activity Programs | Created active spaces for inspire physical activity | Outcome Evaluation | Evaluation report |
| (88) | My Health for Life | Evaluation 1 | My Health for Life (MH4L) program is a state-wide, integrated risk assessment and lifestyle modification program. The program has been developed and led by Diabetes Queensland and implemented by the Health Alliance as part of the Government’s Action Plan for a Healthier Queensland. This program is a free healthy lifestyle program helping Queenslanders to live and age well. | Queensland, 2017-2020 | Physical activity | A face-to-face group-based lifestyle modification program,  Telephone health coaching,  Online support. | Process Evaluation | Evaluation Report |
| (89) |  | Evaluation 2 |  |  |  |  |  |  |
| (90) |  | Evaluation 3 |  |  |  | Workplace Adapted Program, Aboriginal and Torres Strait Islander programs, |  |  |
| (98) | Participation in Community Sport and Active Recreation program (PICSAR) | N/A | VicHealth set up participation in the Community Sport and Active Recreation program (PICSAR) and works with local communities to increase involvement in physical activity, encourage those currently not participating in sport and active recreation to participate, especially Kooris, culturally and linguistically diverse communities, women, older people and people aged 12–25 years and develop partnerships within communities to create more sport and active recreation opportunities. | Victoria - Regional Sports Assemblies, 2002- Continuing | Sports and active recreation | Building capacity and developing partnerships | Process Evaluation | Evaluation Report |
| (99) | Community Street Soccer Program | N/A | The Community Street Soccer Program (CSSP) is an initiative designed to use soccer as a tool to engage and support homelessness, marginalized and disadvantaged communities. Since 2007, The Big Issue has given over 11,300 Australians opportunity to improve their health and positively change their lives. The program aims to promote social inclusion, health, and well-being through regular soccer games and training sessions. | National, 2007- Continuing | Soccer | Weekly soccer matches Training sessions Social activities | Process Evaluation | Evaluation Report |
