# Supplementary material for: Scoping review to assess the reach, effectiveness, and impact of government-funded, population-based physical activity initiatives in Australian adults
Source: Front Sports Act Living. 2025 Oct 10;7:1633086. doi: 10.3389/fspor.2025.1633086 (PMC12550771; doi:10.3389/fspor.2025.1633086)
Supplement: Supplementary file 7 [file Table7.docx]

**S7 Table - Reach, effectiveness and Impact of the grey literature evaluation reports**

| **Study Reference** | **Physical Activity Initiative** | **Reports’ version** | **Reach** | **Effectiveness** | **Results of the effectiveness**  **[Positive (+), Negative (-), Neutral (0), Not Applicable (N/A)]** | **Impact** |
| --- | --- | --- | --- | --- | --- | --- |
| (53) | Heart Foundation Walking | Heart foundation walking. Active Body Active Brain pilot | HFW Local Coordinators, 2,000 volunteer Walk Organisers, 15 pilot walking groups in NSW, SA, NT, Vic, Tas, and WA joined this pilot program.  Average walking time was 35 minutes ranging from a daily to a weekly walk. | Not reported | N/A | The program was more successful at involving people with dementia living in residential care facilities than involving people with dementia in the general community.  While nothing can cure dementia, walking in a group provides a positive, cost-effective Initiative. Physical activity such as walking can reduce some symptoms of dementia and improves the quality of life, and the relationships people have with family, friends and service providers |
| (54) |  | Heart foundation walking 2016 report | 27,665 participants and 1,263 walking groups were activated at the end of December 2016.  Almost 80% are female | Over 80% of walkers reported that in the last week they participated in 150 minutes or more of moderate to vigorous activity.  The retention rate for participants (individuals) continues to be exceptional, with over 77% active after 6 months and 78% after 3 years participation.  Group retention for physical activity program was 84% after 6 months implementation and 77% after 3 years of the program. | + | The Heart Foundation Walking program has a positive impact on individual health and community  well-being by encouraging regular physical activity, improving health outcomes, and fostering social connections |
| (55) |  | Heart foundation walking 2018 report | 45,858 total participants and 1,242 walking groups were activated at the end of December 2018. | The retention rate for participants (individuals) continues to be exceptional, with over 88% active after 6 months and 81% after 3 years participation.  Group retention rates continue to be exceptional with over 80% active after 6 months and over 50% after 3 years participation.  App Walker retention was lower, and people were more likely to be engaged through the app when challenges are operating.  More than 70% of app users are female | + | Not reported |
| (56) | Queensland Walking Strategy Progression Report 2019-2021 | N/A | Piloted and published walking network planning guidance.  Worked with local governments to prioritise and fund improvements for walking.  Raised awareness about road rules that affect people walking.  Partnered with Queensland Walks to facilitate the new Queensland Walking Alliance, Queensland Walks Month and a resource hub | The percentage of trips made by walking increased from 9% to 10.1%  The number of trips under 1 km where people walked increased from 58.5 per cent to 60.2 per cent (based on data for South East Queensland) | + | The Queensland Walking Strategy was significant in promoting walking as a healthy and accessible activity.  The number of pedestrian fatalities and hospitalised casualties decreased from 8.6 per 100,000 population to 6.7 per 100,000 |
| (57) | Queensland state of cycling report 2019 | 2017-2019 report | Expanded principal cycle network, which now identifies more than 10,400 kilometres of network across Queensland, covering more than 99% of the state’s population | The proportion of Queenslanders who ride a bike at least once a year has remained unchanged since 2017 | 0 | Not reported |
| (59) | Queensland state of cycling report 2022 | 2020-2021 report | Since 2019, built another 91 km of bike riding infrastructure along principal cycle routes through the Queensland Government's Active Transport Investment Program**.** | Almost 20% (1 in 5) of Queenslanders rode a bike at least once in a typical week.  The proportion of bike riders who are female increased by 5% between 2017 and 2021.  The 2021 Brisbane Valley Rail Trail Festival of Cycling attracted almost 350 riders to the region.  More than 700 participants logged more than 313,000 km of bike riding to compete in 'Pedal Queensland' online challenge. | + | The Queensland Cycling Strategy increased the number of people cycling, improving cycling infrastructure, and promoting cycling as a healthy and environmentally friendly activity. |
| (58) | Queensland Cycling Action Plan 2020–2022 - Progression Report 2021 | 2020-2022 progression report | Expanded the Principal Cycle Network (PCN) and covered more locations than ever, with 99.5% of Queensland’s population living in a local government area with a PCN Plan. | Delivered 35 bicycle parking.  Funded and completed 37 projects on highest priority PCN routes delivering 23 kilometres of new cycle network.  Supported to the Brisbane Cycling Festival, which attracted more than 20,000 participants and spectators across its 18 days. | + | Not reported |
| (87) | NSW Get Healthy Information and Coaching Service | 2009 Report | Between 23 February and 31 December 2009, the GHS received 5,548 incoming calls including 3,664 new incoming service calls.   For the same period, 17,098 unique visitors visited the GHS website, an average of 389 visitors per week over the 44-week period | Not reported | N/A | The coaching service is designed to support long-term health goals, helping participants to achieve and sustain a healthy and active lifestyle though engaging physical activity.  The 30-second GHS-specific advertising  was significantly more effective at generating contacts compared to ‘Measure Up’ advertising and compared to GHS advertising that involved the 15-second advertising.  The advertising campaign for the GHS effectively increased awareness and engagement of participant for GHS program. |
| (67) |  | 2009-2013 Report | Since 2009, the GHS has averaged approximately reach of the Get Healthy Service 9,500 calls per year and 75,000 unique visits to the website per year.  Coaching participants (a 29.6% increase, from 67.9% in 2009 to 97.5% in 2013),   Males (an 8.2% increase, from 19.5% in 2009 to 27.7% in 2013), and   Aboriginal participants (a 2.4% increase, from 2.3% in 2009 to 4.7% in 2013). | Undertakes recommended amount of physical activity has increased 34% to 62% from 2009 to 2013. | + | Participants who completed the 6 month coaching program on average lost 3.8 kg, off their waist circumference 5.1 cm.  56% of participants who completed the 6-month coaching program lost between of their original body weight 2.5% - 10%.  Aboriginal participants who completed the 6-month coaching program on average lost 4 kg and made significant improvements to healthy eating and physical activity levels  GHS participants considerably improved their risk of chronic disease (Type 2 diabetics), with more than half (56.0%) losing 2.5-10% of their baseline body weight and a further 8% of participants losing more than 11% of their initial body weight. Further there have been changes in the proportion of participants who are classified as being obese |
| (68) | The story of the Tasmanian Get Healthy Information & Coaching Service | 2009 – 2013 and GHS Tasmania | A total of 295 participants had completed the six-month program on 31 December 2013, approximately 29.7 % of those who enrolled in the program. | Not reported | + | Tasmanian participants who complete the 6-month coaching program on average lose 4.7 kg and 5.5 cm off their waist circumference and, increase participants' total physical activity and walking. |
| (69) | This girl can – Victoria 2018 and 2021 | 2018 Report | 700 Target Audience Rating Points (TARPs) (Metro) and 860 TARPs (Regional) delivered via the TV buy, reaching 74% of the audience at least once.   More than 3 million video views via digital advertising and more than 67,000 reached via cinema.  PR activity resulted in 988 mentions, reaching an audience of 8,248,857, at an advertising equivalent value of more than $2 million Social reach of 133,883 on Facebook and 843,266 on Twitter.  More than 30,000 visits to the website and over 500 organisations registered as Campaign Supporters | More than 285,000 Victorian women were more active after seeing the campaign.  This Girl Can – Victoria inspired 1 in 7 women aged between 18-65 across the state to get active.  37% of culturally diverse women said they got active as a result of seeing the adds compared with 28% of the broader community.  17% of women who saw the ad started a new sport or physical activity or returned to exercise after taking a break. | + | Over three-quarters of women who have seen the ad believe it helped women feel more confident getting active.  Right across Victoria, women are participating in more social, fun, supportive and welcoming activities that are what the This Girl Can – Victoria campaign is all about. |
| (70) |  | 2021 Report | Over 340,000 women aged 18–65 were  inspired to get active as a result of seeing the campaign. | Among women who saw the campaign 1–3 times, 25% went on to do something active.  Among women who saw the campaign more than 8 times, 47% of them were active as a result.  Among women who saw the campaign  through one channel, 21% of them went on to do something active.  Among women who saw the campaign  through multiple channels, 39% of them went on to do something active.  1 Victorian women in 6 aged 18–65 got active after seeing the This Girl Can – Victoria campaign.   52% of women with disability recognised the campaign.  52% of culturally diverse women said they got active as a result of seeing the ad as well as 52% of the non-culturally diverse women.  Four months after the campaign ended, 81%of women who did something active had kept it up. | + | Not reported |
| (71) | Be Active program evaluation highlights Improving physical activity outcomes in local communities | N/A | The program successfully engaged a diverse range of participants across five Victorian local councils, including City of Greater Bendigo, City of Greater Geelong, Knox City Council, and Latrobe. It targeted children, older people, and families, aiming to increase their participation in physical activities | Strengthened Council policy and planning Created and strengthened networks and partnerships  Increased participation in physical activity  and volunteering | + | The Be Active program was significant in fostering healthier lifestyles and enhancing civic engagement through volunteerism  This program contributed to reshaping local systems to create healthier communities and fairer health outcomes |
| (85) | 10,000 Steps Workplaces Evaluation | N/A | 51.4% of the respondents (145/282) did implement 10,000 Steps in their workplaces.  26% of health professionals have engaged with the workplace 10,000 Steps program. | 86.4% of respondents believed that the 10,000 Steps program in addressing physical activity as a component of workplace health/wellbeing/wellness program.   81.6 of respondents believed the 10,000 Steps resources and programs increase physical activity and/or steps for those who participated. | + | Providers who had implemented the 10,000 Steps program found it to be a time- and cost-efficient program, which significantly contributed to their professional growth and well-being.   The 10,000 Steps program has been met with resounding approval from providers, with approximately 80% rating the time needed as very low, low, or reasonable. This efficiency is a key factor in the program's potential for success. |
| (86) | Community Activation Program. | N/A | Over the five spaces transformed into five councils, a total of 430 activities were delivered through the Community  More females participated than males in the physical activity program  The program achieved 273,984 unique impressions on social media sites, 5880 visits to project web pages, and 990 articles presented through various media platforms, including print, radio, and online. | Many participants surveyed reported positive shifts in physical activity levels, and the majority (59%) intended to remain more physically active following their participation in local community activation projects. Nine out of ten community members surveyed felt that the space transformed through the program made it easier to be physically active (89%) and more socially connected within their community (93%) | + | The community activation program has inspired more people to participate in physical activity programs and build social connections through these programs.  The projects have resulted in some positive shifts within councils around the value of the activation approaches employed as part of the projects.  The program made a significant impact online |
| (88) | My health for life | Evaluation 1 | 325 participants that completed session 5, 17.2% and 135 participants that completed session 6, 27.4% achieved their goal. | There were significant improvements in the proportion of participants meeting the National Physical Activity guidelines (34.0% at session 1 vs 48.8% at session 5, p=0.002) | + | The Initiative had a positive effect on participant’s health outcomes in the reporting period. Specifically, almost two-thirds of participants lost weight and reduced their waist circumference at session five |
| (89) |  | Evaluation 2 | 8,023 individuals enrolled into the program. A total of 6,564 individuals commenced and A total of 3,939 participants completed MH4L up to session 5 and 2,144 participants completed up to session 6. | Physical activity increased from 223 to 306 minutes per week.  Among session 5 completers, mean physical activity significantly increased from 172 minutes per week at session 1 to 246 minutes at session 5.   Among session 6 completers, mean physical activity significantly increased from 161 to 210 minutes per week | + | Participant lost an average of 0.3% of their body weight. Participant lost an average of 6.7% of their waist circumference. |
| (90) |  | Evaluation 3 | MH4L program has achieved 194,403 risk assessments and 9,051 program completions.  MH4L engaged over 3,000 individual stakeholders. | At session 6, participants met guidelines for physical activity (median of 200 minutes per week) | + | Overall, participants (n=5,287) lost 2% of their body weight (equivalent to an average loss of 1.8 kg) and 2.9% from their waist (equivalent to an average loss of 3 cm)  Many participants adopt long-term changes in their behaviour, such as regular physical activity and healthier eating habits. This can lead to sustained improvements in health over time |
| (98) | Participation in Community Sport and Active Recreation program (PICSAR) | N/A | The PICSAR scheme successfully increased participation in community sports and active recreation across various local communities.  It highlighted the program's ability to engage a diverse range of participants, including those from disadvantaged backgrounds | The scheme was effective in building capacity and developing partnerships  This program promoted the concept of health-promoting organizations and increased opportunities for people to engage in physical activity. | + | The PICSAR scheme was significant in promoting healthier lifestyles and fostering community engagement  This program contributed to reshaping systems to create a healthier and fairer Victoria by ensuring fairer health outcomes and enhancing the local healthcare system |
| (99) | Community Street Soccer Program (CSSP) | N/A | Over 11,300 Australians are engaging in this program | 96% (n=218) of players have reported engaging in physical activity. Forty-three players have suggested that engaging in physical activity was the most significant change they had noticed in their lives.  CSSP helped them improve their fitness as a result (98%, n=217) | + | This Program helps participants build confidence, improve physical fitness, and develop social skills, contributing to their overall well-being and integration into society |
