# Supplementary material for: Scoping review to assess the reach, effectiveness, and impact of government-funded, population-based physical activity initiatives in Australian adults
Source: Front Sports Act Living. 2025 Oct 10;7:1633086. doi: 10.3389/fspor.2025.1633086 (PMC12550771; doi:10.3389/fspor.2025.1633086)
Supplement: Supplementary file 8 [file Table8.docx]

**S8 Table - Characteristics of Grey Literature – Strategy and Actions**

| **Study Reference** | **Name of the strategy and action, Jurisdiction and Initiative years.** | **Summary of the program** | **Physical Activity Type** | **Intervention Type** | **Document Type** | **How will measure or evaluate** |
| --- | --- | --- | --- | --- | --- | --- |
| (60) | Queensland Cycle Strategy 2011–2021 | The Queensland Cycle Strategy 2011–2021 was a comprehensive plan aimed at promoting cycling across the state. The Queensland Cycle Strategy 2011–2021 target is to get more people to cycle more often for school, work, recreation, shopping and social trip. Furthermore, the Queensland Cycling Strategy 2017–2027 sets the strategic direction for cycling in Queensland over the next decade. It aims to support cycling as a convenient, enjoyable, and healthy way to travel and socialize, especially as the population grows. The strategy focuses on building and connecting cycling infrastructure to encourage more people to ride. The Queensland Cycling Action Plan 2020-2022 outlines practical actions to encourage more cycling across the state and | Cycling | Strategy Implementation and actions  Investment in technology | Strategy | A state-wide monitoring framework - a State of Cycling in Queensland Report to be published every two years. |
| (61) | Queensland Cycling Strategy (Re-release) 2017–2027 |  |  | Strategy Implementation Cycling education programs Build and maintain  infrastructure |  | Observations and reporting |
| (62)  (63)  (64) | Queensland Cycling Action Plan 2017-2019  Queensland Cycling Action Plan 2020-2022Queensland Cycling Action Plan 2023–2025 | The Queensland Cycling Action Plan 2017–2019 was part of the broader Queensland Cycling Strategy 2017–2027. |  | Action Implementation | Action Plan | Not reported |
| (65) | Queensland Walking Strategy 2019-2029 | The Queensland Walking Strategy 2019–2029 aims to make walking an easy choice for everyone, every day over the next 10 years. Through the Queensland Walking  Strategy 2019–2029, the Queensland Government will work with partners to ensure their communities are green, liveable and walkable. The Action Plan for Walking 2022–2024 is the second action plan under the Queensland Walking Strategy 2019–2029. It outlines practical actions to create more walkable communities and encourage more walking. | Walking | Strategy and action implementation | Strategy Document and Action Plan | Actions in each two-year Action Plan for Walking will be guided by data, research and evidence |
| (66) | Queensland Action Plan for Walking 2022-2024 |  |  |  |  | Not reported |
| (72) | Strategy A strategy to increase walking for transport in Victoria 2010 -2020 | The Pedestrian Access Strategy sets out the Victorian Government’s vision for a more pedestrian-friendly transport system for Victorians. The aim of the strategy is to encourage more Victorians to walk, especially for short trips. The strategy establishes broad policy principles and the first steps to guide the Victorian Government’s investment in walking over the next 10 years – including infrastructure, planning and design, safety and behaviour change programs. | Walking | Strategy implementation | Strategy Document | Periodic evaluation and ongoing monitoring |
| (73) | City of Melbourne - Walking plan (Victoria) 2014-17 | The purpose of the Walking Plan is to highlight the contribution that walking makes to the municipality, while laying out a practical plan to improve the city’s walking network and encourage more walking. The walking plan aims to increase the number of walking trips in 2030 by 63% from 2009 levels. | Walking | Establishes principles for planning walking in the city | Document of plan | Not reported |
| (74) | Walking and Cycling Strategy Victoria - Shire of Macedon Ranges, 2014-2024 | Walking and Cycling strategy 2014 respond to the high value placed on walking and cycling opportunities by the community by providing clear priorities and guidelines for future action. The primary purpose of the Strategy is to provide Council with strategic direction on ways to increase participation in and improve the supportive infrastructure and resourcing for walking and cycling in the shire over the next ten years. | Walking and Cycling | Strategy implementation - infrastructure and resourcing for walking and cycling | Strategy Document | Annual review (short term evaluation) Comprehensive Review (after 5 years) |
| (75) | Victoria's Trails Strategy 2014–2024 | The purpose of Victoria’s Trails Strategy 2014-2024 is to provide a whole-of government approach and clear vision regarding the planning, management and promotion of trails in Victoria. The vision is for Victoria to be recognised as a leading trail-based destination that provides a diverse range of quality trail experiences for visitors, while strengthening the State’s economy and improving the health, wellbeing and lifestyle of the community. | Walking, cycling, mountain biking, horse riding. | Strategy implementations | Strategy Document | Annual review - Annual report cards. |
| (76) | Victorian Cycling Strategy - Victoria 2018-2028 | The Victorian Cycling Strategy 2018-28 sets out a vision for the future of cycling in the state and a pathway to deliver it. The strategy’s vision is Victoria will increase the number, frequency and diversity of Victorians cycling for transport. This strategy aims to transform cycling over the next decade by setting out the strategic basis to increase cycling for transport | Cycling | Strategy implementation | Strategy Document | Collect data and other information about  cycling and cyclist |
| (77) | Make your Move Greater Dandenong Physical Activity Strategy – Victoria 2020-2030 | The Make Your Move Greater Dandenong Physical Activity  Strategy 2020-2030 has been developed and endorsed by the  council to guide the provision of services, programs, and  infrastructure over the next 10 years. Its goal is to increase the physical activity levels of our community. Through this strategy, the council aims to empower residents to lead more active and healthy lifestyles. | Physical activity  - Walking  -Sports  -Cycling  -Active recreation | Strategy implementation   -Awareness  -Infrastructure facilities | Strategy Document | Annual evaluation and long-term evaluations |
| (78) | Maribyrnong City Council Walking Strategy, Victoria 2023-2033 | The Maribyrnong City Council Walking Strategy 2023-2033 aims to transform Maribyrnong into a more walkable and liveable city. The vision of this strategy is transforming Maribyrnong into a great place for all to walk and contribute to zero greenhouse gas emission travel behaviour. The Strategy aligns with existing policy frameworks, including the Maribyrnong Planning Scheme, Integrated Transport Strategy (MITS), and Council’s Climate Emergency Action Plan. | Walking | Strategy Implementation and actions | Strategy Document | Annual Evaluation |
| (79) | Healthy Parks Healthy People, South Australia, 2016-2026 | Healthy Parks Healthy People SA is a nature-based approach to promoting and protecting population health and wellbeing. It is guided by a vision to ensure that all South Australians are connected to nature and recognise it as an integral component to their health and wellbeing. As the second generation of this program evolves:   Help those South Australians who don’t visit parks and access nature to overcome whatever is holding them back from doing so.  Encourage South Australians who already use parks and access nature to become more regular visitors to gain further health and wellbeing benefits. | Walking | Program Implementation   - Awareness  - Green space for physical activity  - Access | Farmwork | Not reported |
| (80) | Physical Activity in Nature Action Plan, South Australia 2021-2024 | This Action Plan is a key initiative of Healthy Parks Healthy People SA (HPHP SA) - a nature-based approach to promoting and contributing to population health and wellbeing. It is guided by a vision and mission to ensure that all South Australians are connected to nature and recognise nature as an integral component to their health and wellbeing. | (72)Walking and Cycling | Action Implementation | Action Plan | Not reported |
| (81) | Cycling Strategy for South Australia 2022-2023 | Cycling Strategy for South Australia 2022-2023 aim to make cycling a part of everyday life for South Australians and encourage people of all ages and abilities to enjoy the benefits of riding. The purpose of the Strategy is to:   Take advantage of the riding boom   Increase the number of people riding  Promote better infrastructure for riding  Support bike education for children and new riders   Prioritise strategic investment in cycling infrastructure | Cycling | Strategy Implementation | Strategy Document | Not reported |
| (82) | Walking SA Strategic Plan, South Australia 2021 – 2025 | The Walking SA Strategic Plan 2021-2025 aims to encourage  more South Australians to walk more often, promoting  physical health, mental well-being, and environmental benefits. | Walking | Strategy Implementation and actions | Strategy Document | Not reported |
| (83)  (84) | South Australian Walking Strategy 2022-2032 South Australia Walking-Strategy SA Action Plan 2022-2025 | The Strategy is a 10-year blueprint and is accompanied by a  separate action plan that will be reviewed every three years.  Its delivery will fulfil the vision of more South Australians walking more often, all ages, all abilities. The strategy focuses three types of walking: walking for transport, walking for recreation and sport and walking for health and wellbeing. | Walking | Strategy Implementation and actions | Strategy Document and  Action plan | Evidence based action and evaluation. |
| (91) | Tasmania’s Plan for Physical Activity 2011–2021 | Tasmania’s plan for physical activity 2011–2021 is a long-term plan for all of Tasmania. Prepared by the Premier’s Physical Activity Council, in consultation with many other Tasmanians interested in physical activity, it sets a unifying direction and framework for action. The vision of this plan is all Tasmanians experience and enjoy the many benefits of regular physical activity. | Physical activity | leadership and advocacy Infrastructure facilities Sharing information Supporting and expanding programs | Physical activity plan | Annual reports and three-year evaluations |
| (92) | Walk WA: 2007 - 2020 A Walking Strategy and action plan for Western Australia - Sport and recreation | A Walking Strategy for Western Australia 2007 – 2020 (Walk WA) has been developed to assist the community and all organisations and agencies involved in walking throughout Western Australia. Walk WA aims to improve the health and wellbeing of every Western Australian. | Walking | Strategy and action implementation | Strategy Document | Annual reports and three-year reviews |
| (93) | Bushwalking NSW - Strategic Plan NSW 2021-2026 | Bushwalking NSW considers that NSW & ACT should be a premier bushwalking and outdoor recreation destination of Australia, and that our people should enjoy optimal health and wellbeing through community engagement in nature. The vision of Bushwalking NSW is More people walking in nature more often and mission is advocacy and support for, and promotion of, more, safe, inclusive and extensive club-based bushwalking and related outdoor activities in nature. | Walking | Strategy Implementation | Strategy Document | Not reported |
| (94) | Walking Strategy and Action Plan city of Sydney, New South Wales, 2015-2030 | The Walking Strategy and Action Plan for the City of Sydney outlines our approach to creating a more walkable city. It focuses on improving wayfinding, street networks, safety, and public spaces to encourage walking. | Walking | Strategy and action implementation | Strategy and action plan | Annual Reports and five year reviews |
| (95) | Western Australia Hiking Strategy Bushwalking and trail running in Western Australia 2020 - 2030 | The WA Hiking Strategy is a groundbreaking initiative in Western Australia is designed to enhance safe and sustainable hiking experiences for Australians and visitors. This strategy identifies the potential social, cultural, economic, environmental, health and well-being outcomes for the State and articulates a direction for realising these. | Hiking (Bushwalking and trail  running) | Strategy Implementation and actions | Strategy Document | Data collection form quantitative and qualitative methods |
| (96) | New South Wales Healthy Eating and Active Living Strategy 2022-2032 | The NSW Healthy Eating and Active Living Strategy 2022-2032 is a comprehensive plan by the New South Wales (NSW) Ministry of Health. It continues their investment in health and wellness for the people of NSW, with a focus on preventing overweight and obesity. The strategy outlines steps to achieve better health outcomes for all, emphasizing healthy eating and active living. Collaboration between NSW Health, partner organizations, and agencies is key to supporting the health and well-being of the community over the next decade. | Physical activity   Walking  Cycling  Sports | Strategy implementations with prioritise the actions | Strategy Document | Evaluation for Short term, medium and long term  outcomes |
| (97) | Active living for all 2017-2019. A Framework for Physical Activity in Western Australia | Active Living for All 2017-19 represents the strategic direction for increasing and improving opportunities for physical activity and reducing sedentary behaviour in Western Australia. The overarching aim of the framework is to increase physical activity levels and improve opportunities for all Western Australians to participate in all forms of physical activity. The vision for Western Australia is to be the most active state in Australia. This framework was based on key outcome areas of active people and active places. | Physical activity | Strategy implementation | Physical activity framework | Not reported |
| (100) | Community Wellbeing Plan 2014 – 2020 – Darwin | The purpose of the Community Wellbeing Plan 2014 - 2020 is to set direction for Council policy, partnerships and practice to ensure community health and wellbeing is supported within the Darwin Municipality. This plan was developed as part of the Federal Government's Healthy Communities Initiative aimed at delivering community based physical activity and healthy eating programs, as well as developing local policies that support healthy lifestyles. | Sports and recreation Walking | Develop actions for wellbeing | Document of Plan | Community satisfaction with recreation and leisure facilities Bicycle network annual count |
| (101) | Active Transport Strategy - New South Wales, 2022-2028 | The purpose of the Active Transport Strategy is to double active transport trips in 20 years. This Active Transport Strategy draws on the Future Transport Strategy and its vision for walking, bike riding and personal mobility This Strategy provides longer term ambitions accompanied by five-year priority moves to guide planning, investment and priority actions for active transport across NSW. | Walking Bike riding Cycling | Strategy Implementation with prioritise the actions | Strategy Document | Evaluate the strategy and progression |
