# Supplementary material for: Scoping review to assess the reach, effectiveness, and impact of government-funded, population-based physical activity initiatives in Australian adults
Source: Front Sports Act Living. 2025 Oct 10;7:1633086. doi: 10.3389/fspor.2025.1633086 (PMC12550771; doi:10.3389/fspor.2025.1633086)
Supplement: Supplementary file 9 [file Table9.docx]

**S9 Table – Strategies and Actions of Government Funded Physical Activity Initiatives in Australia – Grey Literature**

| **Study Reference** | **Name of the strategy and action** | **Priority Areas** | **Principles** | **Actions/Strategies for physical activity** |
| --- | --- | --- | --- | --- |
| (60) | Queensland Cycle Strategy 2011–2021 | Building safe, direct and connected cycle networks  Growing a cycling culture  Creating cycle-friendly communities  Developing a cycling economy | Not reported | Building safe, direct and connected cycle networks  Building connected cycle networks, developing cycle network plans, making cycling a part of all government infrastructure projects.  Growing a cycling culture  Supporting travel behaviour change to boost cycling, encouraging active school travel, providing information and wayfinding, promoting cycling and community education, supporting cycling events, engaging cycling change champions, ensuring road rules and legislative frameworks support cycling.  Creating cycle-friendly communities  Integrating cycling into planning and development, including end-of-trip facilities in development, delivering a safe cycle network  Developing a cycling economy  Supporting cycle tourism, supporting the bicycle industry, supporting recreation and sports cycling  Building and connecting infrastructure to grow participation   - Building connected networks for cycling, physically separating cycleways, - Finding your way, arriving at your destination, Integrating cycling in the transport network. |
| (61) | Queensland Cycling Strategy 2017–2027 (Re-release) | Building and connecting infrastructure to grow participation  Encouraging more people to ride  Sharing our roads and public spaces  Powering the economy  Using research and data in decision making | Not reported | Building connected networks for cycling, physically separating cycleways,  Finding your way, arriving at your destination, Integrating cycling in the  transport network.  Encouraging more people to ride  Educating new riders, promoting cycling and its benefits,  Supporting the choice to cycle, offering incentives to ride.  Sharing our roads and public spaces  Road rules that work for all, sharing our roads, managing speed to improve safety, Regulating Road space and road use.  Powering the economy  Investing in cycle tourism, Supporting bike business.  Using research and data in decision making  Understanding cycling through research, Collecting and sharing data |
| (62) | Queensland Cycling Action Plan 2017–2019 | Planning for walkable communities and places  Building connected, comfortable and safe walking environments for all  Encouraging more people to walk as part of their ‘everyday’  Working together to deliver for walking | Not reported | Encouraging More People to Ride: Promoting the benefits of cycling as a  healthy, enjoyable, and convenient mode of transport.  Support cycling-related projects, individuals or groups through programs  Support cycling events  Collaborate across Queensland Government to encourage walking and cycling, particularly for transport  Review available research to inform strategies to increase physical activity including cycling and walking  Investigate opportunities  Support creation of more cycle-friendly workplaces  Investigate feasibility of running an electric bicycle trial |
| (63) | Queensland Cycling Action Plan 2020-2022 |  |  | Develop a fun and engaging bike riding campaign  Support bicycle riding events  Support delivery of the Brisbane Cycling Festival  Support community driven projects  Explore opportunities with the private sector to provide electric bicycles (e-bikes) to allow employees to experience using e-bikes for work travel and to understand how e-bikes influence travel behaviour. |
| (64) | Queensland Cycling Action Plan 2023-2025 |  |  | Support community organisations to deliver bike riding road safety education initiatives  Support to deliver localised communication campaigns to increase awareness and the use of new bike riding infrastructure  Support to deliver local programs and events to encourage more  Queenslanders of all ages and abilities to ride bikes, including e-bikes  Support community advocacy for bike riding through initiatives  Research recent Australian and international practices that incentivise bike riding and publish findings |
| (65)  (66) | Queensland Walking Strategy 2019-2029  Queensland Action Plan for Walking 2022-2024 | Planning for walkable communities and places  Building connected, comfortable and safe walking environments for all  Encouraging more people to walk as part of their ‘everyday’  Working together to deliver for walking | Not reported | Planning for walkable communities and places  Planning walkable communities, Planning walking precincts and connected networks, integrating walking with public transport and other travel choices, Designing facilities for all ages and abilities and a range of environmental conditions  Building connected, comfortable and safe walking environments for all  Making roads and precincts safe, building connected and comfortable walking networks, further developing our walking tracks and trails  Encouraging more people to walk as part of their ‘everyday’  Developing a culture that supports walking, Building and supporting walking habits for life  Working together to deliver for walking  Work in partnership to prioritise walking in government policy, investing in walking, using evidence to guide decision-making about walking |
| (72) | Pedestrian Access Strategy A strategy to increase walking for transport in Victoria 2010 | Walking for transport  Health and recreation | Walking should be a viable first choice of transport for short trips and a component of longer trips.  All areas of government have a responsibility to support and encourage better pedestrian access.  Initiatives to increase walking should reflect the needs of all Victorians regardless of age or ability.  Pedestrian access to public transport needs to be easy.  Walking should be considered in all urban planning,  land use and transport development. | Encourage people to walk by changing attitudes and behaviour  Collaborate to improve provision for walking  Create pedestrian-friendly built environments, streets and public spaces  Increase the safety of walking  Continue integrating walking with public transport |
| (73) | City of Melbourne - Walking plan (Victoria) 2014-17 | Not reported | Priority access, Safety, access for all abilities,  Planning for future growth, Creating attractive walking Environments,  Permeability (ability to cross streets)  Reducing delay to pedestrians. | Planning: amending the Melbourne Planning scheme to improve the walking environment.  Street management: changing traffic signal operation to reduce delays to pedestrians; increasing the number of pedestrian streets and shared zones; and improving legibility and way finding.  Capital works: extensive master planning; access around tram and bus stops; and increasing the number of road crossing. |
| (74) | Walking and Cycling Strategy Victoria - Shire of Macedon Ranges, 2014-2024 | Guidelines and standards Connections between towns Connections within towns Tourism trails Support infrastructure Programs and services Marketing and communication | Creating walking and cycle friendly environments and values.  Progressively improving provision of walking and cycling environments.  Opportunities to maximise participation in walking and cycling will be encouraged.  Further development of partnerships and collaboration with relevant agencies and community groups will be critical to achieving the vision, goals and values.  Priorities and staged implementation of improvement initiatives will reflect community needs and organisational capacity | Actions within existing resourcing: actions requiring implementation by Council within existing operational and capital resources (including within ongoing funding provided for implementation of the Shire-wide Footpath  Plan).    Priority actions: combination of capital, planning and operational actions requiring Council lead to implement and are considered a priority and achievable within the life of this plan.  Aspirational actions: prioritised list of actions requiring Council lead that are to be implemented as opportunities arise (e.g. external grants and funding, additional Council resources, developer contributions and/or other partnerships as appropriate). |
| (75) | Victoria's Trails Strategy 2014–2024 | Investment  Development  Awareness  Experience  Marketing | Principles for investment  Experience: trail user profile, quality, access, safety  Benefits: Economic, Environmental and social  Sustainability: Demand, Landowners and/or land managers, development costs, long-term commitment of resources, natural and cultural value, planning requirements and Strategic imperative | Provide a strategic framework for trail investment  Support effective planning,  Development and maintenance of trails  Provide high quality information on trails  Create better trail experiences  Market trails. |
| (76) | Victorian Cycling Strategy - Victoria 2018-2028 | Investment  Safer  Lower Stress  Better connecting network  Cycling corridors  More Inclusive | Not reported | Investing in a safer, lower-stress, better-connected cycling network, prioritising strategic cycling corridors.    Making cycling a more inclusive experience:  The strategy aims to encourage a broader range of people to cycle. By improving infrastructure, safety, and accessibility, it seeks to make cycling an attractive choice for everyone, regardless of age, ability, or background. |
| (77) | Make your Move Greater Dandenong Physical Activity Strategy – Victoria 2020-2030 | Not reported | Affordability: Fees and charges  Cost to Council: affordability to provide physical activity facilities to the community  Equitable and Inclusive: fair and equal access to physical activity  opportunities and resources  Partnerships: Effective and mutually beneficial partnerships  Systems-Approach: Multiple policy actions, strategies, programs and services  Universal Design | Empower the community to be physically active, supporting them to make choices through easy-to-understand information  Support and facilitate opportunities for our community to be physically active  Be a leader in supporting physical activity participation and advocate for our community to improve health, social and wellbeing outcomes through increased participation in physical activity  Create environments that encourage and support our community to be physically active  Use evidence to guide and drive decisions about how to increase participation in physical activity and improve the health of our community |
| (78) | Maribyrnong City Council Walking Strategy, Victoria 2023-2033 | Prioritizes and promotes walking across the municipality | Not reported | Promote a healthy and liveable municipality where walking is preferred for local trips  Create a walking network that all people are confident using.  Strengthen the presence of walking within streets, places and transport interchanges.  Create a walking culture that celebrates the recreational role of walking. Ensure walking is inclusive to all genders and abilities, to be a primary driver in elevating community health and wellbeing. |
| (79) | Healthy Parks Healthy People, South Australia, 2016-2026 | Not reported | Not reported | Promoting physical activity in nature (Action plan in development)  Increase the number of people in South Australia who pursue physical activity in nature  Enhance the availability and accessibility of green and blue public spaces  that support physical activity  Increase equity in access to physical activity in nature - to increase the opportunities for vulnerable and disadvantaged communities to experience and be physically active in nature |
| (80) | Physical Activity in Nature Action Plan, South Australia 2021-2024 | Not reported | Not reported | More people being active in nature.  Action topics - Awareness and motivation, Confidence and competency and Activity opportunities that lead to habit forming.  Quality spaces that support physical activity in nature.  Action topics - Provision and distribution, achieving quality accessible spaces, Achieving partnerships  Equitable access to engage in physical activity in nature  Action topics - Advocate for equity, Research and evidence, Equity capacity building |
| (81) | Cycling Strategy for South Australia 2022-2023 | Information and  Promotion  Greenways  Bicycle Boulevards  Integrating Cycling  into Major Projects  Major Events &  Programs  Trails and Elite  Sport | Not reported | Inclusive Cycling  Cycling for everybody, everyday  Accessible Cycling  A modern, convenient and safe cycling network  Integrated Cycling  Land use, transport and public transport planning working together  Enjoyable Cycling  Cycling tourism, sport and recreation opportunities |
| (82) | Walking SA Strategic Plan, South Australia 2021 – 2025 | Not reported | Not reported | Goal 1: More walking for recreation, transport, and health  Strategies  Grow walking participation through programs, walking clubs and walking SA  supporters  Support the planning of walkable communities and environments  Provide community information and lead annual walking events  Goal 2: Effective and viable functioning of Walking SA  Promote the brand and value of Walking SA  Raise and consolidate the profile of Walking SA through partnership development  Demonstrate strong governance, financial stability and sustainability |
| (83)  (84) | South Australian Walking Strategy 2022-2032 South Australia Walking-Strategy SA Action Plan 2022-2025 | Plan walkable neighbourhoods, towns and cities  Build connected, safe and pleasant walking environments for all  Create a South Australian walking culture   - Walking for transport - Walking for recreation and sports - Walking for health and Wellbeing | Not reported | Plan walkable neighbourhoods, towns and cities  Plan for walkable places  Plan for integrated public transport and walking  Plan for universally accessible walking facilities for all ages and abilities  Build connected, safe and pleasant walking environments for all  Create connected and pleasant walking networks  Balance the needs for all travellers on our roads and footpaths  Reinvigorate our main streets and local neighbourhoods  Enhance our walking tracks and trails  Create a South Australian walking culture  Educate, market and promote walking  Realise the economic and tourism value of walking in South Australia |
| (91) | Tasmania’s Plan for Physical Activity 2011–2021 | Not reported | Not reported | Goals  Become a community that values and supports physical activity  Involve leadership and advocacy  Create built and natural environments that enable and encourage physical activity  Involve modifying building standards and codes of practice, tailoring the design of buildings and infrastructure, making sure planning schemes adequately zone and protect open spaces, and marketing and promoting what is on offer.  Develop partnerships that build and share knowledge and resources  Involve sharing information, supporting locally relevant research, and extending the use of existing infrastructure for physical activity.  Increase opportunities for all Tasmanians to be physically active where they  live, work and play  Involve supporting and expanding programs that are working well, and systematically analysing barriers and finding solutions. |
| (92) | Walk WA: 2007 - 2020 A Walking Strategy for Western Australia - Sport and recreation | Not reported | Diversity  Equity  Access and availability  Quality  Efficiency and effectiveness  Advocacy  Adaptability | To provide strategic management, advocacy and coordination of Walk WA  Work with government, stakeholders, policy and advocacy  To promote walking.   Highlight the physical, mental, and social benefits of walking  To improve the places where people walk  Develop and maintain walking-friendly environments  To improve safety and security of places where people walk  Ensure the walkers safe and protections  To implement and expand programs and services which provide opportunities for walking  Establish the walking programs and share the success of the initiatives. Create the partnership with other organisations |
| (93) | Bushwalking NSW - Strategic Plan NSW 2021-2026 | Not reported | Not reported | NSW & ACT: A Premier Bushwalking Destination:  Advocate for 500 kilometres of new walking tracks within 5 years.  Support iconic long-distance track initiatives.  Retain existing walking routes and tracks.  Advocate for at least 17% of NSW as intact natural areas protected by  National Parks by 2030.  Initiate public discussions on granting public access to walking on private  land.  Better health for more people through bushwalking  Increase the number of people regularly experiencing outdoor adventures  in natural areas through affiliate clubs  Enhanced stakeholder relations  Foster positive relationships with stakeholders.  Continuous operational improvement  Ensure efficient and transparent governance |
| (94) | Walking Strategy and Action Plan city of Sydney, New South Wales, 2015-2030 | Make walking quick, convenient and easy  Make walking inviting and interesting  Make walking safe and comfortable  Create a strong walking culture | Not reported | Make walking quick, convenient and easy  Priority for people walking, Complete networks and Wayfinding  Make walking inviting and interesting  Lively streets, Attractive and interesting places  Make walking safe and comfortable  Road safety, Personal security and Comfort  Create a strong walking culture  Changing attitudes and supporting travel choice, and school and workplace strategy implementation. |
| (95) | Western Australia Hiking Strategy Bushwalking and trail running in Western Australia 2020 - 2030 | Not reported | Access  Consumer focus  Consistency  Sustainability  Evidence base  Innovation  Community benefits  Visitor economy contribution  Engagement  Quality trail  Aboriginal participation  Environment and culture | Maximise participation outcomes  Encourage participation at all levels and capitalise on the benefits of trail running and bushwalking.  Grow the visitor economy  Grow visitation by increasing the reputation of Western Australia’s new and existing experiences, including commercial enterprises, events and activities.  Sustainable trail development and maintenance  Develop and manage sustainable hiking opportunities in Western  Australia.  Effective governance and advocacy  Increase and optimise the management and resourcing of hiking activities in  Western Australia through strong governance and advocacy. |
| (96) | New South Wales Healthy Eating and Active Living Strategy 2022-2032 | Support to closing the gap through initiatives co-designed with Aboriginal and Torres Strait Islander people.  Embed non-stigmatising approach to promote healthy eating and active living | Not reported | Prevention programs and services to support healthy eating and active living  Routine advice on healthy eating and active living as part of clinical care  Healthy food and built environments to support healthy eating and active living  Social marketing to support healthy eating and active living behaviour change |
| (97) | Active living for all 2017-2019. A Framework for Physical Activity in Western Australia | Strengthening public policy,  Providing appropriate environments and programs (active places and active people),  Increasing public motivation and understanding,  Promoting partnerships,  Transitioning research into practice. | Local planning processes  Identifying funding opportunities and providing information to guide  Policy and program development | Well planned and designed environments supporting, encouraging and enabling active living  Strategies consisted with infrastructures, access for physical activity and active transport.  Initiatives that promote positive behaviour change and opportunities  to participate in active lifestyles.  Strategies evidence-based behaviour change program, campaign, education, access and active play opportunities. |
| (100) | Community Wellbeing Plan 2014 – 2020 - Darwin | A healthy and active community  Families, friends and community in Darwin The natural world, parks & gardens Diverse cultures and history of Darwin A sustainable lifestyle Opportunity, affordability and equity | The natural, built, social and economic environments on the health and wellbeing of the community | Provide exercise equipment in the CBD to cater for increased apartment living.  - Encouraging an active lifestyle for all  - Supporting access to affordable, fresh produce |
| (101) | Active Transport Strategy - New South Wales, 2022-2028 | The Strategic Cycleway Corridors Program, in each of the six cities, will deliver safe, connected, and continuous cycling networks linked to local cycle networks. | Not reported | Enable 15-minute neighbourhoods  Deliver continuous and connected cycling networks  Provide safer and better precincts and main streets  Promote walking and cycling and encourage behaviour change  Support our partners and accelerate changes |
